# Are the Targets of the Nature Restoration Regulation Achievable at a Regional Scale? An Analysis of Natura 2000 Sites on the Island of Sardinia

**DOI:** 10.1007/s00267-025-02242-x

**Published:** 2025-08-22

**Authors:** Michele Defraia, Erika Bazzato, Michela Marignani

**Affiliations:** 1 Department of Life and Environmental Sciences, University of Cagliari, Cagliari, Italy; 2 University School for Advanced Studies IUSS, Pavia, Italy; 3 Project e.INS - Ecosystem of Innovation for Next Generation Sardinia, Cagliari, Italy

**Keywords:** Prioritization, Italy, Habitat, Ecosystem restoration, Conservation targets

## Abstract

The Nature Restoration Regulation (NRR) aims to restore 20% of degraded terrestrial and marine ecosystems across Europe by 2030. One of the initial provisions states that, by 2030, Member States should prioritize the restoration of natural ecosystems within Natura 2000 sites, emphasizing the urgency of assessing the conservation status of habitats in these areas. We selected Sardinia as a case study to evaluate the feasibility of the NRR at the regional level. The Natura 2000 sites in Sardinia cover a comparable percentage of territory (18.87%) to the national (19.38%) and European level (18.6%). Additionally, Sardinia’s insularity, high biodiversity levels, and low population density make it an ideal model for testing restoration strategies. Using official Natura 2000 data provided by the Italian Ministry of the Environment, we assessed the potential for restoration for each habitat within each site based on the conservation status values. The results indicated that coastal ecosystems were the most endangered. However, their limited distribution meant that their restoration would have a modest impact on achieving the NRR target. In contrast, forest and shrub habitats, which were more widely distributed, emerged as the main contributors to the restoration goals. Conducting this study at a regional level allowed us to provide actionable recommendations for management practices to be locally adopted. Our findings confirmed that restoration efforts confined to Natura 2000 sites alone would be insufficient to meet the NRR targets, underscoring the need to implement additional restoration measures in agricultural, urban, and other natural and semi-natural areas.

## Introduction

Biodiversity loss, habitat degradation, and climate change effects are substantial enough to justify the urgent implementation of active measures aimed at reversing current environmental degradation and promoting long-term ecosystem resilience (Díaz et al. 2019; Brondízio et al. 2021).

The latest results of the Habitats Directive Art. 17 Reports (EC 2020) suggest that, despite the number of regulations aimed at environmental safeguards implemented in Europe over the years (i.e., the Habitats Directive (HD), Birds Directive (BD), Water Framework Directive, and Marine Strategy Framework Directive), efforts to preserve natural environments remain insufficient.

Within this European context, the Nature Restoration Regulation (hereafter NRR) aims to switch the focus of environmental capital management from “conservation” to “restoration”(Hering et al. 2023). Initially slated for approval by the European Council in March 2024, the NRR faced delays due to a lack of consensus among Member States. It was ultimately approved on June 17, 2024, after incorporating a series of compromises to establish more achievable targets and mitigate political opposition.

The main focus and target of the law are defined as follows: “This Regulation establishes a framework within which Member States shall put in place effective and area-based restoration measures with the aim to jointly cover, as a Union target, throughout the areas and ecosystems within the scope of this Regulation, at least 20% of land areas and at least 20% of sea areas by 2030, and all ecosystems in need of restoration by 2050 (Ch. I, Art. 1, pt. 2) (EC 2024).

Key strategies for achieving these goals are detailed according to various ecosystem types. The NRR specifies measures for terrestrial, marine, urban, and agricultural ecosystems, species-related habitats, and relevant parameters and indicators for evaluating restoration effectiveness, ensuring a scientifically grounded approach (Cliquet et al. 2024). For example, a sub-target for marine ecosystems requires putting in place restoration measures on at least 30% of the marine habitats’ total area by 2030 (Ch. II, Art. 5); sub-targets for urban ecosystems require ensuring that there is no net loss of urban green space area and urban tree canopy cover by 2030 and establishing an increasing trend starting in 2031 (Ch. II, Art. 8); and a sub-target for agricultural ecosystems requires increasing trends in at least two indicators from among the butterfly index (Swaay et al. 2013), stock of organic carbon and share of high-diversity landscape features in agricultural land by 2030 (Ch. II, Art. 11).

In the case of natural terrestrial ecosystems, it is specified that by 2030, restoration measures shall be implemented on at least 30% of the area covered by HD habitats, primarily in Natura 2000 sites (Ch. II, Art. 4, pt. 1). This implies that an analysis of the conservation status of the habitats within the Natura 2000 sites is a prerequisite. Natura 2000 is a network of protected areas, established to preserve the most valuable natural habitats and species across Europe. The sites constituting the Natura 2000 network are designated under the Bird and Habitat Directives.

Given that target percentages must be met across the entire Union territory, there is a risk of an unequal distribution of restoration responsibilities among Member States (Hoek 2022). Although Chapter III of the NRR includes provisions for National Restoration Plans to facilitate each Member State’s contribution to the overall restoration effort, no specific targets are set for individual States.

Even though the targets are intended to be accomplished on a European scale, identifying the areas for restoration necessarily requires an analysis of the territory at the local level. In this phase, it is possible to test the feasibility of applying the same targets to the regional scale beforehand.

We focused on the ecosystem restoration in Natura 2000 areas through an analysis of the degree of conservation of habitats: assessing the degree of conservation of these areas offers valuable insights into which ecosystems are in greatest need of restoration interventions.

By working at the regional scale, it is possible to provide preliminary suggestions for future management practices to the responsible entities.

This paper aims to evaluate the feasibility of accomplishing the NRR targets at the regional level by taking the island of Sardinia (Italy) as a pilot site. Sardinia is an administrative region of Italy, classified under the Nomenclature of Territorial Units for Statistics as a level 2 region with the code ITG2. By giving priority to habitats within Natura 2000 areas, we focused on two specific objectives: i) to estimate the proportion of Sardinia’s territory that could potentially be subjected to ecosystem restoration measures (meeting the 20% target, based on Ch. I, Art. 1, pt. 2) and ii) to estimate the proportion of the total area of habitat types within Sardinian Natura 2000 network that could be subjected to ecosystem restoration measures (meeting the 30% target, based on Ch. II, Art. 4, pt. 1).

Adopting a bottom-up approach enables the systematic planning and implementation of ecological restoration efforts while promoting an equitable allocation of responsibilities in meeting the European Union’s restoration targets. This regional-scale analysis can serve as a replicable model for spatial planning and environmental management across comparable European and International regions.

## Materials and Methods

### Study area

The island of Sardinia, covering an area of 24,090 km², is the second-largest island in the Mediterranean basin. Its population is nearly 1.6 million residents, resulting in a population density of 64.97 residents per km². This density is relatively low compared to the Italian national average of 196 residents per km² and the European average of 115 residents per km².

The topography is mainly hilly, with an average elevation of 350 m above sea level, low mountains primarily located in the eastern area and a few plains, such as the Campidano plain, which is the largest of the island and is located in the southwest area.

According to the 2008 Sardinia Land Use Map (www.sardegnageoportale.it), approximately 58% of the island is covered by natural areas, 38% by agricultural areas, 2% by water bodies, and 3% by urban areas. Its landscapes mainly consist of forests (~21%), shrubs and garrigue (~24%) and grasslands, which, together with mixed agricultural areas and herbaceous pastures, occupy almost 40% of the territory (Salis et al. 2021).

The climate is typically Mediterranean, with hot and dry summers and relatively mild and rainy winters; the mean annual temperatures range from 7 to 17 °C, with maximum temperatures often exceeding 30 °C during the summer (Salis et al. 2021). From a bioclimatic perspective (the interrelation between climate and the distribution of living organisms, particularly plant communities), the latest studies, based on the classification of Rivas-Martínez et al. (2011), have identified two macrobioclimates (Mediterranean pluviseasonal oceanic and Temperate oceanic), four classes of continentality (from weak semihyperoceanic to weak subcontinental), eight thermotypic horizons (from lower thermomediterranean to upper supratemperate) and seven ombrothermic horizons (from lower dry to lower hyperhumid), which together resulted in 43 isobioclimates (Canu et al. 2015; Bazzato et al. 2021).

Sardinia hosts 128 Natura 2000 sites, which encompass 18.9% of its land area. This percentage is close to the national average, with Natura 2000 sites covering 19.4% of Italy’s territory, and the European average, where these sites cover 18.6% of the territory. The average number of Natura 2000 sites per Italian region is 125, which is consistent with Sardinian ones (MASE 2024).

In addition to the Natura 2000 network, Sardinia has several other protected natural areas established under regional or national laws. These include four natural regional parks (RAS 2024), and three national parks (MASE 2022), although one of them, “Parco Nazionale del Golfo di Orosei e del Gennargentu”, remains inoperative and lacks official management (MASE 2015). The region also has nine Ramsar sites (MASE 2021), six marine protected areas (MASE 2021), and 41 public forests (RAS 2025), which are protected and managed by the Regional Agency for Forests under Regional Law no. 8 of April 27, 2016 (Lai et al. 2017) (Table 1).

**Table 1:** Natural protected areas in Sardinia outside Natura 2000 network

Type of area	Number of areas	Institution	Establishment
Natural Regional Park	4	Autonomous Region of Sardinia	Regional Law no. 31/1989
National Park	3	Italian Ministry of the Environment and Energy Security	Decree of the President of the Italian Republic
Ramsar site	9	Convention of Ramsar	Ministerial decree
Marine Protected Area	6	Italian Ministry of the Environment and Energy Security	Ministerial decree
Public forest	41	Autonomous Region of Sardinia	Regional Law no. 8/2016

The heterogeneous landscapes, bioclimates variability, and the significant number of protected sites in Sardinia reflect the high natural value of the region, establishing it as a recognized biodiversity hotspot within the Mediterranean basin (Médail 2017; López-Alvarado et al. 2020; Thompson 2020).

Due to its insularity — being self-contained and not affected by factors from contiguous areas —its low population density, which may result in reduced ecosystem restoration costs (Zhao et al. 2023), and the presence of several protected areas, the region of Sardinia serves as an ideal model to test the feasibility of the NRR target.

### Definition of the Nature Restoration Regulation terrestrial habitats

To define which ecosystems are to be restored, the NRR considers all terrestrial habitats listed in Annex I of the Habitats Directive and classifies them into six groups (hereafter NRR Groups): 1) wetlands (coastal and inland), 2) grasslands and other pastoral habitats, 3) river, lake, alluvial and riparian habitats, 4) forests, 5) steppe, heath and scrub habitats, and 6) rocky and dune habitats.

By categorizing the habitats into these groups, the NRR promotes a structured approach to assessing and implementing restoration measures. Each group comprises specific habitat types that share common ecological characteristics, allowing for targeted restoration strategies that address their unique needs and conservation challenges.

To test the feasibility of the NRR objectives at the island scale, the original targets of the NRR for the European territory have been scaled to the study area level.

To achieve these targets, each country should first quantify the total area of habitat types, as part of drafting a national restoration plan (Art. 15). According to Cazzolla Gatti et al. (2024), the current best available spatial resolution for the habitat distribution map in Italy is 10 km. Given the current lack of fine-resolution mapping of Habitats Directive (HD) habitats at both the national level in Italy and the regional level in Sardinia, we chose to constrain the analyses to the data pertaining to the Natura 2000 sites. Therefore, only the areas of HD habitats within Natura 2000 sites were used to evaluate the two objectives.

The full list of terrestrial habitats in the NRR framework is available in the Supplementary Materials (Supplementary Table S1).

### Collection of Natura 2000 Network data

Member States are required to submit periodic evaluations of Natura 2000 sites to the European Union, specifically through Standard Data Forms (SDFs), to meet the targets set by the Habitats Directive (HD). These Standard Data Forms provide data in a structured and comparable format, ensuring consistent monitoring and reporting. Each SDF is updated annually for every Natura 2000 site and includes administrative information, such as site registration and geographic location, and data related to ecological factors and the degree of conservation of ecosystems.

The “degree of conservation” evaluates the conservation condition of a habitat or species within a specific site. A notable sub-criterion within the degree of conservation is the “restoration possibility,” which directly aligns with the goals of this project.

Each habitat within a site is documented with its surface area and a general site assessment, which includes evaluations of data quality, representativity, and relative surface.

The degree of conservation of ecosystems within Natura 2000 sites in Sardinia was evaluated using official data from the Italian Ministry of the Environment and Energy Security (Ministero dell’Ambiente e della Sicurezza Energetica – MASE). For this study, we collected the SDFs for the years 2021, 2022, and 2023 and we compiled them into a comprehensive database. We used the most up-to-date information available for each site. Additionally, we assigned a label to distinguish the different site types (ST) across the database. This detailed and methodical approach ensures the reliability of the data used to assess the feasibility of achieving the NRR targets in Sardinia, while also ensuring the repeatability of the process.

Understanding the distinctions between the various types of Natura 2000 sites (ST) is crucial for conducting a coherent comparison of the data. The Natura 2000 network consists of Special Areas of Conservation (SACs), Sites of Community Importance (SCIs), and Special Protection Areas (SPAs). SACs and SCIs are designated under the Habitats Directive, with SCIs differing in that they are pending an official act assuring the conservation measures necessary to become SACs. SPAs are designated under the Birds Directive and, consequently, their implementation is more focused on species-related measures.

To obtain non-redundant results, we divided Natura 2000 sites into three site types according to their designation directive, conservation focus and overlapping status: Birds Directive Site Type (BD_ST), which consists only of SPAs; Habitat Directive Site Type (HD_ST), which consists of SACs and SCIs; and Birds Directive/Habitat Directive Site Type (BD/HD_ST), which consists of SACs and/or SCIs that perfectly coincide with SPAs. This means that some unique sites, with a unique identification code, are both SAC and SPA simultaneously (Table 2).

**Table 2:** Site types according to their designation, Natura 2000 area types and conservation focus

Site type (ST)	Designation	Area type	Conservation focus
BD_ST	Birds Directive	SPA	Species
HD_ST	Habitats Directive	SAC SCI	Habitats
BD/HD_ST	Habitats Directive and Birds Directive	SAC/SCI spatially overlapped with SPA	Habitats and species

Analysing the data by SPAs and SACs can lead to duplication of some sites, while discriminating them by site type prevents this redundancy because BD_ST, HD_ST, and BD/HD_ST are mutually exclusive. We performed the following analyses on habitats exclusively by site type: this approach ensures that results are non-redundant and accurately reflect the distinct characteristics and conservation requirements of each site type within the Natura 2000 network in Sardinia. However, BD/HD_ST does not include sites that are exclusively SPAs or SACs, which in some cases may partially overlap.

### Definition of Habitat Conservation Status Triggering Restoration Measures

**Fig. 1:**
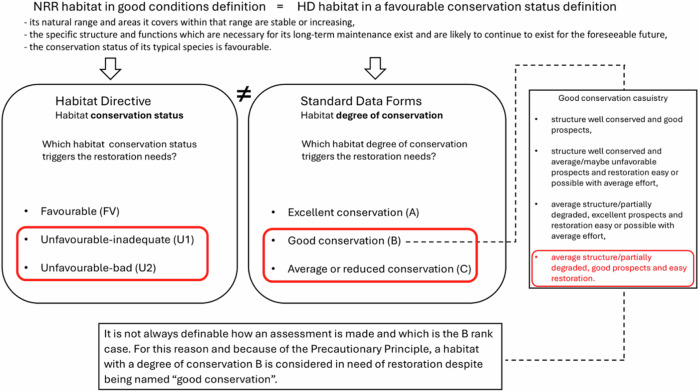
Concept map illustrating the determination of habitat conservation status that triggers the need for restoration measures under the NRR, based on the evaluation systems

To identify habitats requiring restoration under the Nature Restoration Regulation (NRR), it is essential to clearly define the concept of conservation status. We adopted a logical process, outlined in Fig. 1, which involved: i) Reviewing the legal definitions of conservation status as established by the Habitats Directive (HD) and the Standard Data Forms (SDFs); ii) Comparing classification systems used in the HD and SDFs; iii) Interpreting how these systems translate into restoration obligations under the NRR.

According to the NRR (Ch. II, Art. 4, pt. 1), Member States are required to restore habitats listed in Annex I that are not in “good condition,” as defined by the HD’s criteria for “favourable conservation status” (Ch. I, Art. 3). A habitat achieves favourable conservation status when its natural range and area are stable or increasing, its structure and functions are sufficient for long-term maintenance and the status of its typical species is favourable (EC 1992). Habitats not meeting these criteria are considered in need of restoration.

The HD classifies conservation status into three categories: Favourable (FV), Unfavourable-Inadequate (U1) and Unfavourable-Bad (U2). In contrast, the SDFs adopt the degree of conservation classification method, with three levels: A (Excellent), B (Good) and C (Average or Reduced).

This assessment is based on three sub-criteria (EC 2018): Structure conservation, rated as excellent (I), well-conserved (II), or average/partially degraded (III); Function conservation (prospects), rated as excellent (I), good (II), or unfavourable (III) and Restoration feasibility, categorized as easy (I), moderate (II), or difficult/impossible (III). The final degree of conservation is assigned hierarchically: Degree A is assigned if the structure is excellent (I), or if it is well-conserved (II) with excellent prospects (I); Degree B includes habitats with well-conserved structure and good/average prospects (restoration feasible), or with average structure and excellent/good prospects; Degree C is applied when neither A nor B criteria are met (see Table 3).

**Table 3:** Classification of habitat degree of conservation in Natura 2000 according to the Standard Data Forms (SDFs), based on three sub-criteria: structure conservation, functional conservation, and restoration feasibility, with classification levels A, B, and C

Degree of conservation	Sub-criteria
A (Excellent)	- Excellent structure (I), regardless of other criteria. - Well-conserved structure (II) and excellent prospects (I).
B (Good)	- Well-conserved structure (II) and good prospects (II). - Well-conserved structure (II), average prospects (III), and easy (I) or moderate (II) restoration. - Average/partially degraded structure (III), excellent (I) or good (II) prospects, and easy (I) or moderate (II) restoration.
C (Average or Reduced)	- Any other combination of structure, prospects, and restoration possibilities not included in A or B.

In cases where the representativity of a habitat is rated as D (non-significant presence), no further assessment is required, and the degree of conservation is automatically assigned as 0 (unclassified).

For the purpose of identifying habitats in need of restoration under the NRR, habitats with degree of conservation A are considered in “good condition” and do not require restoration, analogous to HD’s “Favourable” (FV) status while habitats with a degree of conservation C are clearly in need of restoration, similar to U1 or U2 categories under the HD.

Habitats with a degree of conservation B present ambiguity, as they may range from “structure well conserved and good prospects independent of the grading of the third sub-criterion” and “average structure/partially degraded, good prospects and easy restoration”.

Since both conservation status and degree of conservation assessments lack precise and unified guidelines, their definition varies across Member States and often relies on expert judgment (Alberdi et al. 2019). As a result, it is not always clear how an assessment is made. For this reason, we included habitats with a degree of conservation rated as B in the category of habitats in need of restoration, following the Precautionary Principle (EU 2016).

### Habitat surface analysis

We conducted statistical analyses on the entire dataset. In addition, specific assessments were carried out exclusively for the Habitat Directive Site Type (HD_ST). These areas were selected as a model because of their greater importance in terms of extension, number of sites and habitats. Moreover, the designation of HD_ST aligns more closely with the objectives of habitat conservation compared to the Birds Directive Site Type (BD_ST), which is primarily focused on the preservation of avifauna species. This approach prevented double-counting of areas where HD_ST and BD_ST partially overlap, thereby avoiding an overestimation of the surface area.

We analysed the amount (in terms of number of habitats, i.e. habitat records, and surface area), distribution, and state of conservation of Natura 2000 habitats to estimate the surfaces that could potentially be restored in the NRR framework. The land cover area of each site type and conservation degree, as reported in the Standard Data Forms (SDFs), was calculated based on the surface area of individual habitats. We excluded from statistical analysis habitats with an unclassified conservation status (i.e., 0) or no reported surface area. The UTM WGS84 coordinates (longitude and latitude) of the Natura 2000 site centroid of the investigated habitats were used as a parameter to establish the geographical location.

Due to the presence of a few extreme values, i.e. particularly large surface areas associated with forest habitats, we opted for non-parametric statistical tests, which are more robust to non-normal data distributions and less sensitive to outliers. The statistical analysis included the following steps: i) Non-parametric one-way ANOVA: Conducted on three groups representing the surface area of habitats classified under conservation degrees A, B, and C, to evaluate significant differences among HD_ST habitats based on their conservation degree. ii) Non-parametric unpaired t-test: Performed to compare the surface areas of habitats with conservation degree A and those with degrees B and C combined, representing habitats in need of restoration. iii) Non-parametric one-way ANOVA: Conducted using three variables (habitat surface area, site longitude and site latitude) to assess whether geographical location is significantly associated with differences in surface area. iv) Dwass–Steel–Critchlow–Fligner post hoc test: Applied for pairwise comparisons among habitat surface area, geographic location, and conservation degrees to identify specific differences between groups. v) Spearman correlation analysis: Employed as a complementary step to explore monotonic relationships between habitat surface area and the geographical coordinates (longitude and latitude) of the sites, independently from the ANOVA outcomes. Statistical analysis was conducted using GraphPad Prism version 8.0.2 (GraphPad 2019).

Furthermore, we drafted 4 maps to visualize how the habitats’ surface areas are distributed across the HD_STs considering the different conservation degrees. For each conservation degree and for conservation degrees B and C combined, we calculated 3 class sizes (i.e. Small, Medium and Large) obtained by Jenks natural breaks classification methods. This allowed us to visualize the location of the most suitable potential restoration areas according to the management recommendation. The elaboration of the maps was performed with QGIS software version 3.40.2 (QGIS 2025).

## Results

### Amount, Distribution and Conservation Status of Habitats in Different Site Types

**Fig. 2:**
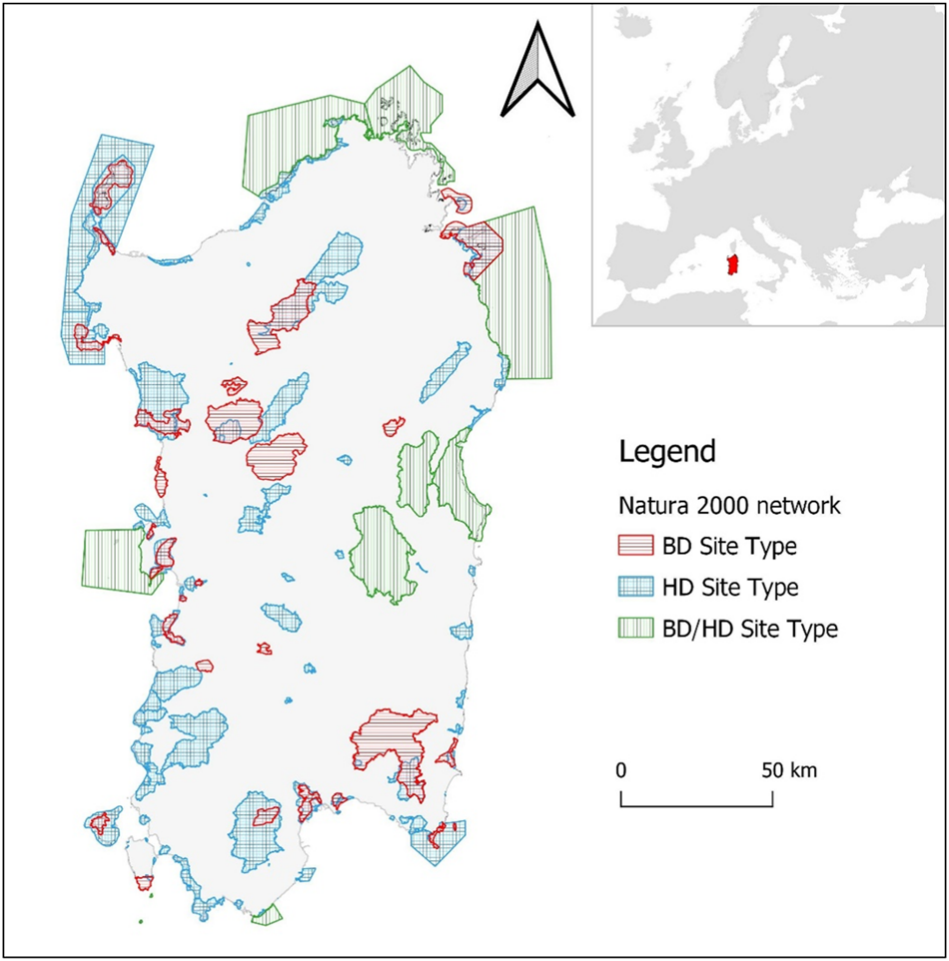
Map of Sardinia Natura 2000 network divided by site type

In Sardinia, there are 49 habitat types within the 31 Birds Directive Site Types (BD_ST), including 44 terrestrial and 5 marine habitats. The 87 Habitat Directive Site Types (HD_ST) host 55 habitat types (50 terrestrial, 5 marine), while the 10 Birds Directive/Habitat Directive Site Types (BD/HD_ST) encompass 49 habitat types (44 terrestrial, 5 marine) (Fig. 2). Regarding habitat records listed in the Natura 2000 site types, BD_ST includes 324 habitat records, HD_ST 1,087, and BD/HD_ST 110. Four sites have no habitat data in their Standard Data Forms. These sites, all of which are 100% marine areas, include one HD_ST site (“Dall’Isola dell’Asinara all’Argentiera”, code ITB013051) and three BD/HD_ST sites (“Da Tavolara a Capo Comino”, code ITB013050; “Da Capo Testa all’Isola Rossa”, code ITB013052; and “Capo Spartivento”, code ITB044010).

The conservation degree values are distributed in similar percentages across the different site types.

Among the 57 terrestrial habitats, “Vegetated sea cliffs of the Mediterranean coasts with endemic *Limonium spp*.” (HD code 1240) has the highest number of habitats with a conservation degree A (11.40%).

“Thermo-Mediterranean and pre-desert scrub” (HD code 5330) has the highest proportion of habitats with conservation degree B (6.96%), while “Annual vegetation of drift lines” (HD code 1210) and “Embryonic shifting dunes” (HD code 2110) have the highest percentages of habitats with conservation degree C (9.09% and 8.73%, respectively).

“Shifting dunes along the shoreline with *Ammophila arenaria* (white dunes)” (HD code 2120), “Pseudo-steppe with grasses and annuals of the *Thero-Brachypodietea*” (HD code 6220), and “Southern riparian galleries and thickets (*Nerio-Tamaricetea* and *Securinegion tinctoriae*)” (HD code 62D0) represent the habitats with the highest proportion of unclassified conservation degree (i.e., 0) (5.49% each).

In BD_ST, conservation degree A is represented most frequently associated with habitat 1240 (11.11%). Conservation degree B is primarily linked to habitat 5330 (9.26%) for BD_ST. Conservation degree C in BD_ST is most represented by “Mediterranean salt meadows (*Juncetalia maritimi*)” (HD code 1410) and “Mediterranean and thermo-Atlantic halophilous scrubs (*Sarcocornetiea fruticosi*)” (HD code 1420) (both with 12.28%). Conservation degree 0 is most common for “*Salicornia* and other annuals colonizing mud and sand” (HD code 1310) (9%) in BD_ST.

In HD_ST conservation degree A is represented the most by habitat 1240, with a percentage of 11.93%. Conservation degree B in HD_ST is primarily linked to habitat 5330 (6.56%) The most frequent habitats associated with conservation degree C in HD_ST include 1210 (8.78%), 2110 (9.27%), “*Crucianellion maritimae* fixed beach dunes” (HD code 2210) (8.29%), and “*Brachypodietalia* dune grasslands with annuals” (HD code 2240) (9.27%). The habitat most commonly linked to conservation degree 0 in HD_ST is “*Quercus ilex* and *Quercus rotundifolia* forests” (HD code 9340) (7.14%).

In BD/HD_ST, conservation degree A is most represented by “Caves not open to the public” (HD code 8310) and 9340, both at 11.54%. Conservation degree B in BD/HD_ST is most common for “Mediterranean temporary ponds” (HD code 3170) (10%). Conservation degree C in BD/HD_ST is most represented by 1210 and 2110, both with a percentage of 15.38%. The habitat representing conservation degree 0 in BD/HD_ST is habitat 2120 (15.38%).

The total cover is 646.65 km^2^ for BD_ST, 1512.80 km^2^ for HD_ST, and 486.29 km^2^ for BD/HD_ST.

**Fig. 3:**
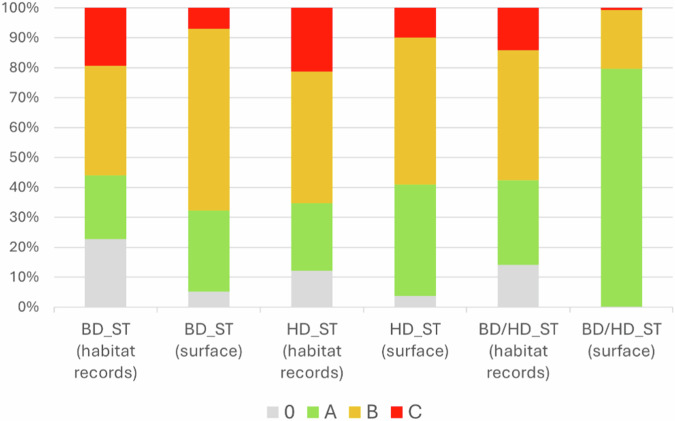
The distribution of conservation degrees across habitat records is similar among different site types, whereas habitat surface areas show notable differences

The distribution of conservation degree values varies among the different site types. Degree B is the most common in BD_ST and HD_ST, whereas degree A is predominant in BD/HD_ST (Fig. 3).

Considering the surface area of the NRR groups, in BD_ST, group 4 (Forests) is the most represented, covering 213.34 km^2^ (190.14 km^2^ with a conservation degree B), while group 6 (Rocky and Dune habitats) is the least represented, with 28.07 km^2^. The habitat with the largest surface area in BD_ST is 9340, covering 128.9 km^2^, followed by 6220 (97.78 km^2^) and “Dehesas with evergreen *Quercus spp*.” (HD code 6310) (95.30 km^2^). The smallest surface area in BD_ST is occupied by “Spartina swards (*Spartinion maritimae*)” (HD code 1320) (0.0243 km^2^).

**Fig. 4:**
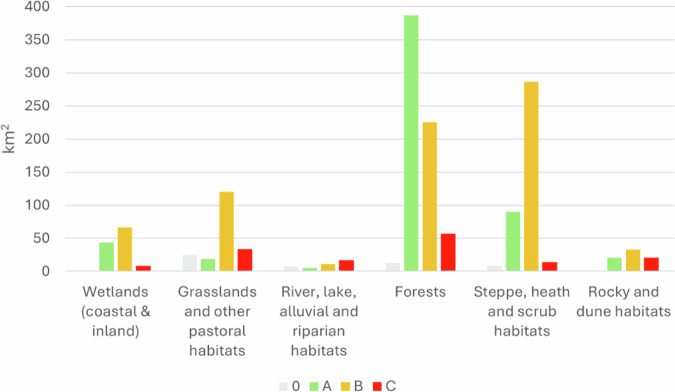
Distribution of the surface area of NRR Groups per different degrees of conservation in Habitat Directive Site Type

In HD_ST, the NRR group 4 (Forests) is the most represented, covering 682.53 km^2^, while group 3 (River, lake, alluvial and riparian habitats) is the least represented, with 41 km^2^ (Fig. 4). The habitat with the largest surface area in HD_ST is 9340, covering 456.23 km^2^, followed by 5330 (188.94 km^2^). The smallest surface area in HD_ST is occupied by “Water courses of plain to montane levels with the *Ranunculion fluitantis* and *Callitricho-Batrachion* vegetation” (HD code 3260), which spans only 0.425 km^2^.

In BD/HD_ST, the NRR group 4 (Forests) is the most represented, covering 231.99 km^2^, while the least represented is group 1 (Wetlands, coastal & inland) with 0.30 km^2^. The habitat with the largest surface area in BD/HD_ST is 9340, spanning 216.9 km^2^, followed by “Arborescent matorral with *Juniperus spp*.” (HD code 5210) (75.44 km^2^), and the smallest area is “Mediterranean salt steppes (*Limonietalia*)” (HD code 1510) (0.002 km^2^).

Among the different site types, Habitats 8310 and “Submerged or partially submerged sea caves” (HD code 8330) are the only ones recorded with surface area values of zero.

Detailed data on habitat records can be found in the Supplementary Material from Supplementary Tables S2–S5, while full data on the distribution of the surface areas across different conservation degrees are provided from Supplementary Tables S6–S8.

### Ecosystems to be Restored in the NRR Framework

In Sardinia, according to the NRR Groups (terrestrial, coastal and freshwater habitats), 57 habitat types were recorded, with the groups “River, lake, alluvial and riparian habitats” and “Rocky and dune habitats” being the most represented, comprising ~44% of the habitat types listed at national level in Italy.

In terms of the number of habitats records, we documented 1321 habitats, with the NRR groups “Steppe, heat and scrub habitats” and “Rocky and dune habitats” being the most represented (Table 4).

**Table 4:** Number of habitat types of Italy and Sardinia and habitat records in Sardinia per different NRR Groups

	Wetlands habitats	Grassland habitats	River, lake, alluvial and riparian habitats	Forests habitats	Steppe, heat and scrub habitats	Rocky and dune habitats
Italy habitat types	18	18	22	33	15	24
Sardinia habitat types	7	6	12	9	9	14
Sardinia habitat record	189	103	172	156	260	441

**Fig. 5:**
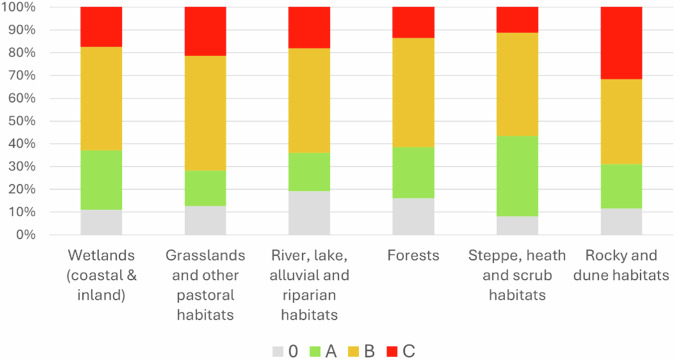
The degrees of conservation of the habitat records have a different distribution among different NRR groups

**Fig. 6:**
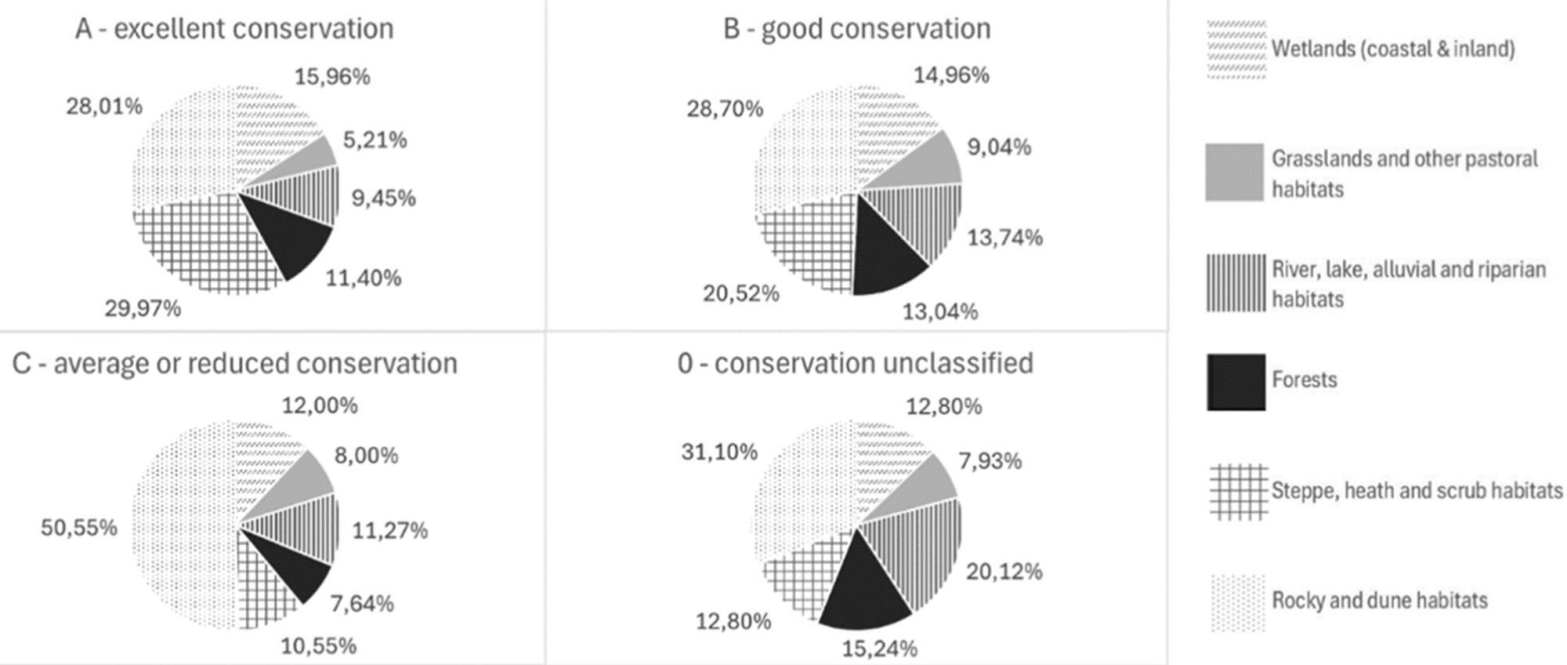
Percentage distribution of habitat records per different degrees of conservation per NRR Groups. Arranged clockwise starting from the top-left: habitats with an excellent conservation degree, habitats with a good conservation degree, habitats with an unclassified conservation degree and habitats with an average or reduced conservation degree

The conservation status vary among different NRR Groups, but B is the most common value (Fig. 5). Steppe, heat and scrub habitats were recorded as the NRR Group with the highest percentage of habitat records with a conservation degree A (29.97%), followed by Rocky and dune habitats (28.01%). The ecosystem with the lowest percentage of habitat records with a conservation degree A is Grassland habitats (5.21%). Rocky and dune habitats is the NRR group with the highest percentages for conservation degree B (28.70%), C (50.55%) and 0 (31.10%). Grassland habitats is the NRR group with the lowest percentages of conservation degree 0 (7.93%) and B (9.04%), while Forests have the lowest percentage of degree C (7.64%) (Fig. 6).

### Surface Area Analysis and Statistical Insights, A Focus on Habitat Directive Site Types

In Habitat Directive Site Type (HD_ST), the habitats with a conservation degree A cover a total surface area of 563.96 km^2^, those with a conservation degree B cover 743.03 km^2^, and those with a conservation degree C cover 149.95 km^2^. Combined, habitats with conservation degrees B and C span approximately 893 km^2^. This means that restoration measures could potentially target 3.7% of Sardinia’s total territory. However, this is insufficient to meet the first target of a 20% threshold.

When considering the proportion of habitats potentially requiring restoration measures, compared to the total habitat area within HD_ST (1512.80 km^2^), the ~893 km^2^ in need of restoration corresponds to 59.02%. This means the second target of a 30% threshold can indeed be achieved.

Focusing on habitat patch dimensions, habitats with a conservation degree A are generally larger than those with a conservation degree B and C (Table 5). A similar trend is evident when examining different NRR groups, particularly in group 1 (Wetlands), group 4 (Forests) and group 6 (Rocky and dune habitats) (Table 6).

**Table 5:** Mean dimension (ha) of the habitats in Habitat Directive Site Type per different degree of conservation

Degree of conservation	Habitats mean coverage (ha) in HD_ST
A	263.53
B	174.83
C	73.87

**Table 6:** Mean dimension (ha) of the habitats in Habitat Directive Site Type in NRR groups per different degree of conservation

NRR groups	Excellent conservation (A)	Good conservation (B)	Average or reduced conservation (C)
Wetlands (coastal & inland)	127.5	102.6	44.7
Grasslands and other pastoral habitats	186.8	342.3	222.3
River, lake, alluvial and riparian habitats	25.9	21.5	77.5
Forests	1546.9	480.0	356.4
Steppe, heath and scrub habitats	142.4	341.5	77.2
Rocky and dune habitats	32.7	23.1	18.1

**Fig. 7:**
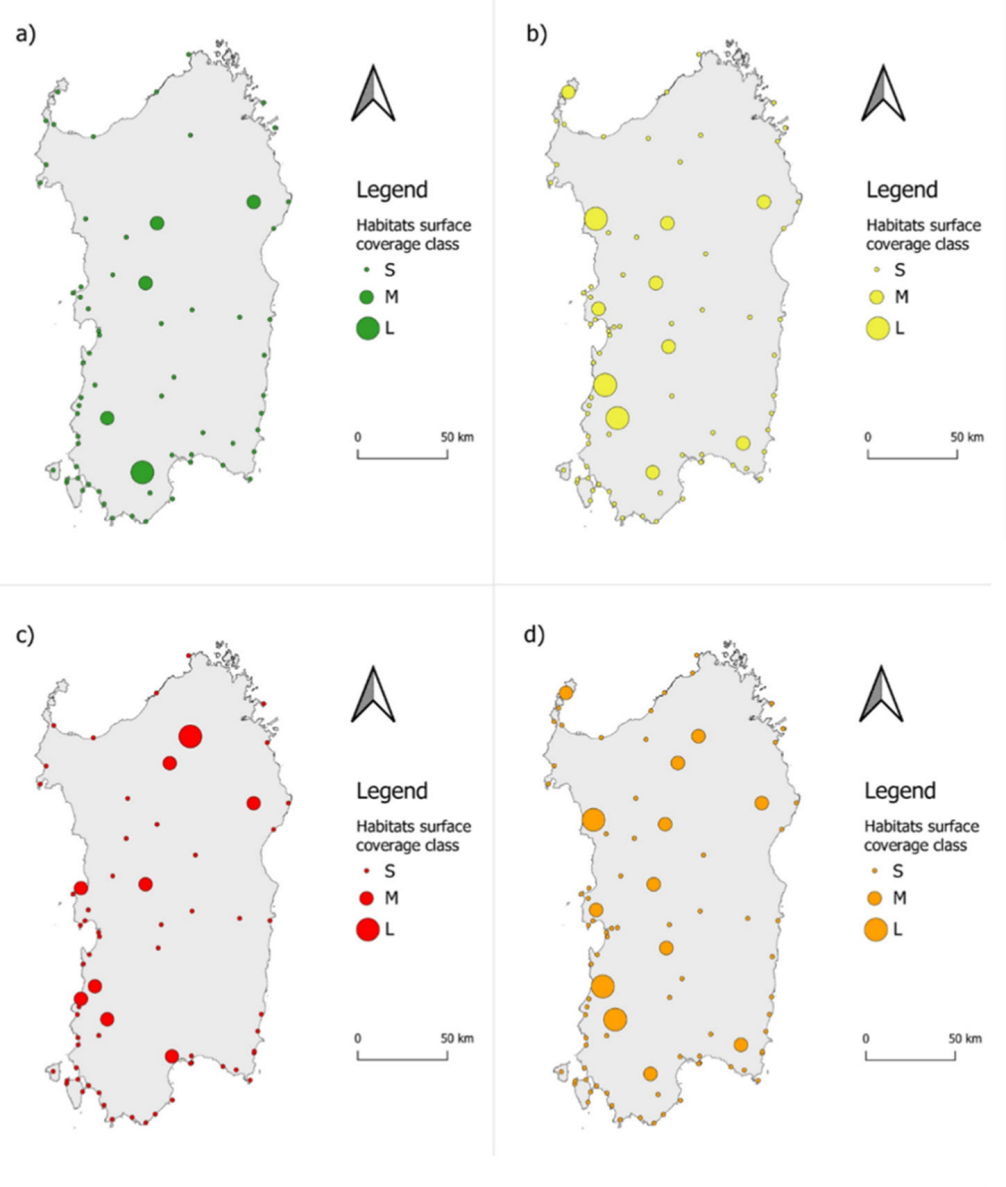
Geographical distribution of habitat surfaces across the Habitat Directive Site Type. The habitat surface coverage classes S (small), M (medium) and L (large) have been obtained by Jenks natural breaks classification method. **a** Habitats with a degree of conservation of A. **b** Habitats with a degree of conservation of B. **c** Habitats with a degree of conservation of C. **d** Habitats with a degree of conservation of B and C together (habitats in need of restoration)

The geographical distribution of habitat surfaces across the HD_STs is shown in Fig. 7, with three size classes for each conservation degree calculated using the Jenks natural breaks classification method. The largest potential areas for intervention are associated with habitats showing conservation degrees B and C (Fig. 7).

We investigated whether there are significant differences between habitats’ surface areas and their degree of conservation.

**Fig. 8:**
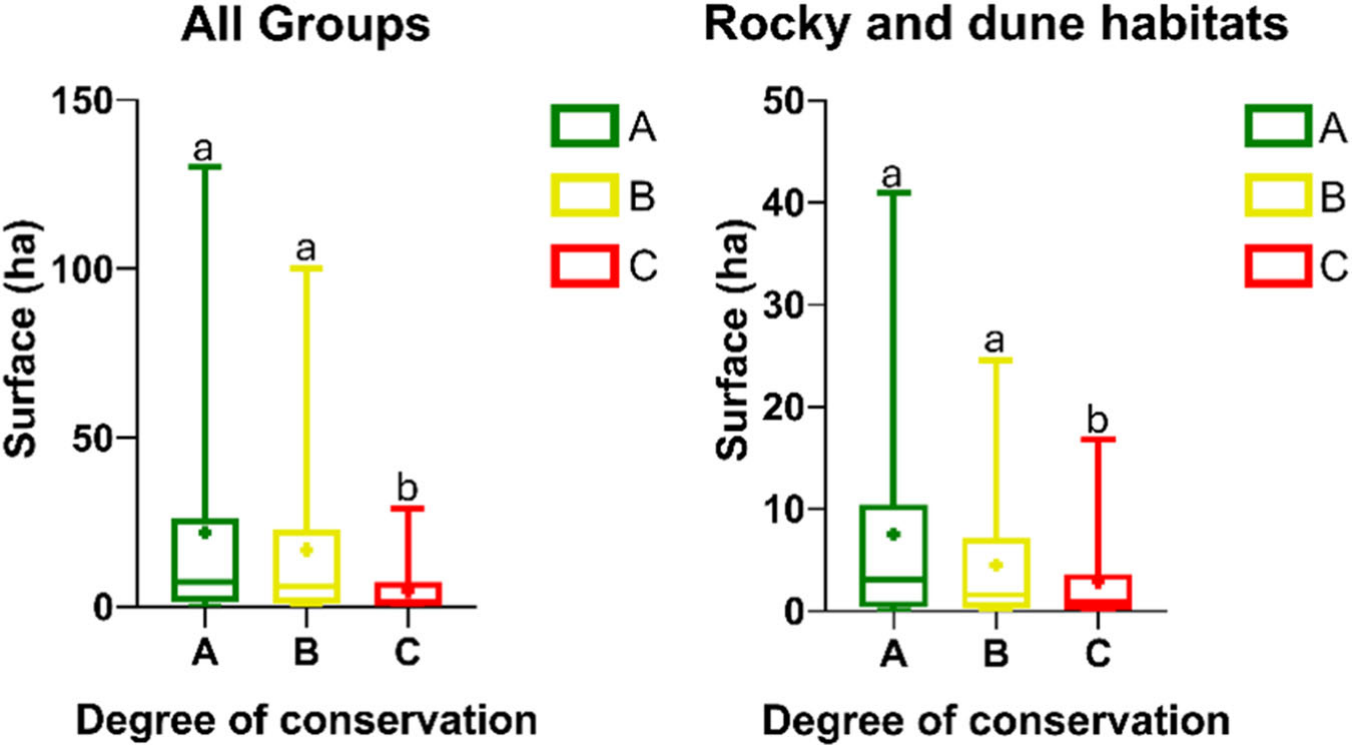
Habitat surface differences between degrees of conservation. Symbol “+” represents the mean value. Lowercase letters highlight significant differences in pairwise comparison

The results of the one-way non-parametric ANOVA test revealed a significant difference in surface coverage among the different conservation degrees (*p* ≤ 0.0001) (Table 7). When the same test was performed for the different NRR groups, a significant difference was observed only within the Rocky and dune habitats group (*p* = 0.0027) (Fig. 8).

**Table 7:** Results of the one-way non-parametric ANOVA test (Kruskal–Wallis test) for habitat surface coverage across the three conservation degrees

	*p* value	Kruskal–Wallis
General	**<0.0001**	28.16000
Wetlands (coastal & inland)	0.3971	1.84700
Grasslands and other pastoral habitats	0.5024	1.37700
River, lake, alluvial and riparian habitats	0.9561	0.08979
Forests	0.5269	1.28200
Steppe, heath and scrub habitats	0.6342	0.91070
Rocky and dune habitats	**0.0027**	11.82000

Values showing significant differences are highlighted in bold

**Fig. 9:**
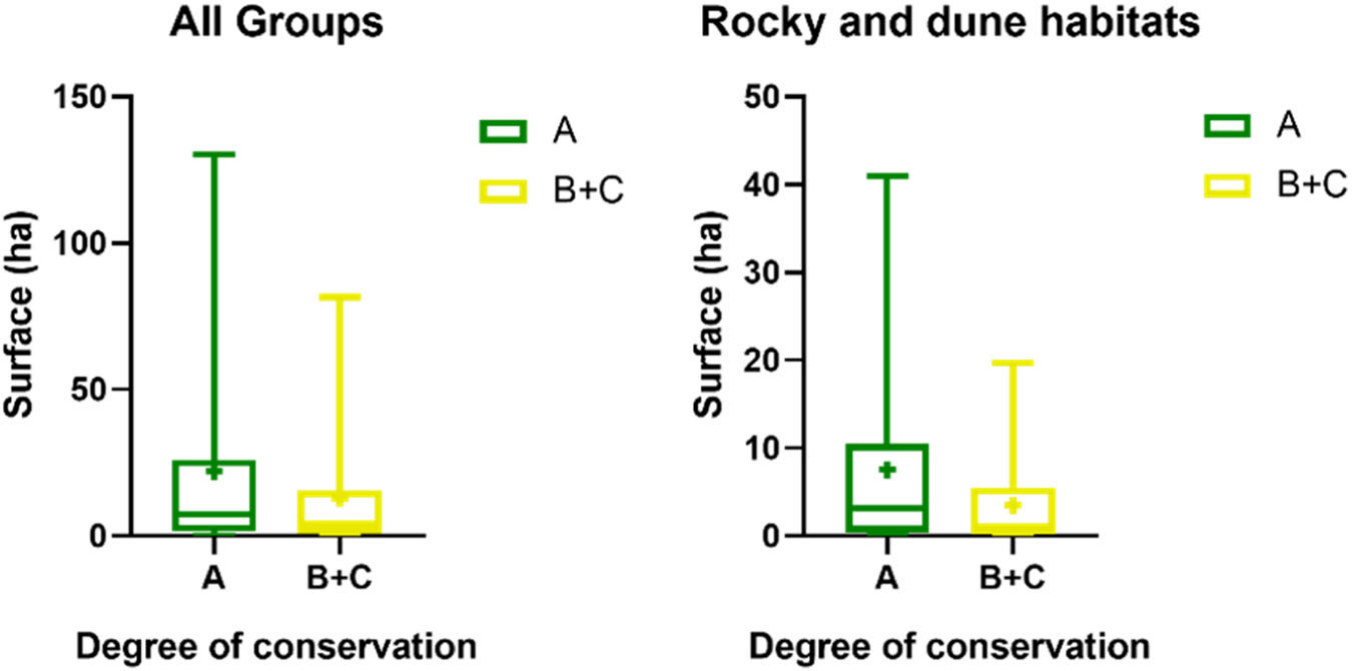
Surface area differences between habitats in a good conservation status (degree of conservation A) and habitats in need of restoration (degree of conservation B and C). Symbol “+” represents the mean value

The results of the non-parametric unpaired t-test, comparing the surface areas of habitats with a conservation degree A to those of habitats with combined conservation degrees B and C (representing in need of restoration), revealed significant differences both in general terms (*p* = 0.0084) and specifically within the Rocky and dune habitats NRR group (*p* = 0.0134) (Fig. 9).

Significant differences were observed between habitat surface areas and their geographic location. A one-way non-parametric ANOVA test revealed a significant difference between habitat coverage and their latitudinal position (*p* < 0.001).

Pairwise comparisons using the Dwass–Steel–Critchlow–Fligner test showed significant differences between conservation degrees A-B and B-C in terms of habitat coverage, and between B-C for the latitudinal parameter (*p* < 0.001).

Furthermore, a positive and significant correlation was identified between the habitat surface area and latitude (Spearman’s coeff. = 0.169; *p* < 0.001).

The complete results of the t-test are reported in Supplementary Materials (Supplementary Table S10), while the full results of the Dwass–Steel–Critchlow–Fligner test are available from Supplementary Tables S11a to S17b.

## Discussion

The present study has specifically focused on terrestrial ecosystems in Sardinia, aligning with the restoration priorities set forth by the NRR. While marine ecosystems also play a crucial role in the broader context of biodiversity conservation, their restoration criteria and priorities differ from those of terrestrial ecosystems, as indicated in Chapter II, Article 5 of the NRR. Therefore, a separate evaluation would be needed to assess the conservation status of marine habitats. In our analysis, we concentrated on Natura 2000 sites within Sardinia, recognizing that these areas are central to the NRR’s objectives. By examining the conservation status of the various habitats in these sites, we identified areas with the highest potential for restoration and the greatest need for intervention.

### Insights into the Assessment of Sardinia’s Habitat Conservation Status

Results show that the coverage of habitats listed in the Habitat Directive Standard Data Forms accounted for ~50% of the total site area, which was considerably lower than the overall site area. Specifically, habitats of interest covered 43.08% of the total area in Birds Directive Site Type, 56.12% in Habitat Directive Site Type, and 50.01% in Birds Directive/Habitat Directive Site Type. This discrepancy could be attributed partly to limitations in detecting all existing habitats within a site, but it also highlighted that a Natura 2000 site is not necessarily entirely occupied by natural areas. A substantial proportion of these sites was found to consist of agricultural land or, in some cases, urban areas, reflecting the ongoing expansion of urban zones within Natura 2000 sites in recent years (Concepción 2021).

The ratio of natural to agricultural areas within Natura 2000 was found to align with that of Sardinia’s overall territory, reinforcing the validity of using Natura 2000 sites as a representative sample to evaluate the application of the NRR on the island.

The prioritization of restoration measures in Natura 2000 areas applies exclusively to natural ecosystems (Ch. II, Art. 4), whereas the restoration of urban and agricultural ecosystems is governed by distinct regulations (Ch. II, Art. 8 and 11), that do not reference Natura 2000. However, agricultural and semi-natural habitats within Natura 2000 hold added value, as High Nature Value (HNV) farmland (sensu Baldock et al. 1993; Beaufoy et al. 1994) considerably overlaps with farmland in these areas (EC 2018). HNV comprehends European areas where agriculture is the dominant land use and supports or is associated with high habitat diversity. For instance, high-diversity landscape features, which are natural or semi-natural elements embedded in agricultural contexts, are proven to be key factors in providing ecosystem services and support for biodiversity (Rubio-Delgado et al. 2024). The HNV concept is explicitly mentioned in the Nature Restoration Regulation for inclusion in the National Restoration Plans (Ch. III, Art. 14, pt. 2), along with the high-diversity landscape features that constitute a specific target to achieve for the restoration of agricultural ecosystems (Ch.II, Art.11, pt.2). Furthermore, any land use changes within Natura 2000 are regulated under the protection regimes of Art. 6 of the Habitat Directive (Möckel 2022). Given these reasons, we propose that restoration measures for agricultural and urban ecosystems within Natura 2000 areas should be aligned more closely with those for natural ecosystems, or at least given differentiated attention compared to areas outside Natura 2000.

The proportion of terrestrial habitats in Sardinia compared to Italy (57 in Sardinia vs. 130 in Italy per the NRR) was consistent with the proportion of total registered habitats observed in prior grey literature (61 in Sardinia vs. 137 in Italy) (Biondi et al. 2009), both approximating 44%. This percentage difference was primarily due to Sardinia being located entirely within the Mediterranean biogeographic region, whereas continental and alpine biogeographic regions are also represented in the rest of Italy.

While many habitats are not exclusive to a single biogeographical region, restoring the greatest possible variability of ecosystems inherently requires involving multiple regions with distinct biogeography and ecology (Fischer and Lindenmayer 2007; Capotorti et al. 2015; Blasi et al. 2017).

Despite the substantial variation in the number of sites across different types of site protection, the variability of the habitat types remained comparable. For instance, Birds Directive/Habitat Directive Site Type (BD/HD_ST) encompassed only 7 sites (3 of which lacked data) but exhibited the number of habitats similar to BD_ST (31 sites) and just 6 fewer habitat types than HD_ST (87 sites). This was consistent with the fact that 4 BD/HD_STs (ITB010008 “Arcipelago La Maddalena”, ITB020014 “Golfo di Orosei”, ITB021103 “Monti del Gennargentu”, ITB022212 “Supramonte di Oliena, Orgosolo e Urzulei - Su Sercone”) fell within 2 National Parks (“Parco nazionale del Golfo di Orosei e del Gennargentu” and “Parco nazionale dell’Arcipelago di La Maddalena”) established for their high naturalistic value of international or national relevance (Lovari and Cassola 1975).

The greater habitat type variability in Habitat Directive Site Type supported its selection for more detailed analysis included in our study.

Within all sites, Rocky and dune habitats (NRR Group 6) were found to have the highest number of records with conservation degree C (i.e., with 441 habitat records), consistent with their status as the most represented group overall. At the same time, these habitats, containing the most degraded areas, also hosted the habitat type with the highest proportion of conservation degree A: habitat 1240, “Vegetated sea cliffs of the Mediterranean coasts with endemic *Limonium spp*.” This habitat, characterized by vegetation of halo-rupicolous species, is intrinsically subject to pressures different from other ecosystems. It mostly consists of highly specialized plants that bear the marine aerosol and live in the cracks of the rocks (Biondi 2007), which are highly limiting factors that reduce interspecific competition. The soil exploitation is barely present, and the main threats to this habitat are rock climbing, invasive alien plants, and landslides (Strumia et al. 2020). These characteristics suggest the ability of these habitats to protect themselves or at least be less prone to degradation.

Steppe, heat and scrub habitats (NRR Group 5), though less representative (260 habitat records), exhibited the highest number of records with conservation degree A. Within this group, habitats such as “Low formations of *Euphorbia* close to cliffs” (HD code 5320) and “Thermo-Mediterranean and pre-desert scrub” (HD code 5330) have the highest number of records with conservation degree A, showing particularly strong conservation outcomes. This could be attributed to the long-term evolutionary adaptation of Mediterranean sclerophyllous plants to drought conditions, which rendered these ecosystems more resilient to seasonal drought events (Bussotti et al. 2014). Despite their overall good state, habitats in conservation degree B (i.e. 5330 as the most representative in HD_ST) were also noted, reflecting methodological approaches that flagged these as degraded enough to necessitate restoration.

Habitat 8310, “Caves not open to the public” (Rocky and dune habitats), faces similar conditions to habitat 1240, as inaccessibility is a defining characteristic that makes it less susceptible to pressure. In the absence of natural or anthropic disturbances, this habitat is expected to remain stable over time (Biondi et al. 2009). However, it requires special attention due to the lack of standardized European guidelines for assessing its conservation status (Weigand et al. 2022).

Coastal habitats, such as habitat 1210 “Annual vegetation of drift lines” and 2110 “Embryonic shifting dunes”, were found to be in poor conservation status, with the highest numbers of records in conservation degree C. These habitats represent the foredune environment (i.e., the area between the shoreline and the dune crests) (Acosta et al. 2009). Habitat 1210 consists only of annual therophytic-halonitrophyl vegetation, bounded seaward by the aphotic zone reached by waves and connected landward to habitat 2110, which is characterized by perennial, geophytic, and hemicryptophytic psammofiles that form “embryonic dunes” (Carranza et al. 2008). These habitats are unsurprisingly in poor health, as coastal ecosystems are among the most threatened ecosystems worldwide (Del Vecchio et al. 2019; Delbosc et al. 2021), with beach tourism — particularly pronounced in Sardinia (Balletto et al. 2019) — being one of the main pressures (Malavasi et al. 2013; Pinna et al. 2022).

Various projects, such as those funded by the EU LIFE Programme, have been and continue to be implemented to safeguard and restore these ecosystems and consolidate the dunes. However, their limited land coverage significantly restricts their contribution to the NRR targets. In Natura 2000 sites, their coverage is generally only a few hectares, with many cases under one hectare. There are only a few exceptions, such as site ITB013019 “Isole del Nord - Est tra Capo Ceraso e Stagno di San Teodoro”, which has a coverage of approximately 180 hectares for each habitat.

Wetland habitats (e.g., 1410 “Mediterranean salt meadows (*Juncetalia maritimi*)”, and 1420 “Mediterranean and thermo-Atlantic halophilous scrubs (*Sarcocornetiea fruticosi*)”) also showed degradation trends in BD_ST, confirming the vulnerability of these ecosystems (Dudgeon et al. 2006; Bolpagni et al. 2018; Perennou et al. 2020). Wetlands (along with peatlands) offer critical ecosystem services (Costanza et al. 2014; Mitsch et al. 2015; Fois et al. 2021), such as carbon stock, food chain support, and pollutant removal, while also helping to mitigate extreme events such as floods (Maltby and Acreman 2011). This makes them an asset that is always worth investment in for restoration. Additionally, since the SPAs are designed to protect avifauna species, managing and restoring wetland habitats ensures the protection of species listed in the Birds Directive (Ferrarini et al. 2021; Fois et al. 2022).

Habitat type 1310 “*Salicornia* and other annuals colonizing mud and sand” has the highest number of records with an unclassified conservation degree (i.e., 0), particularly represented in BD_ST. This is primarily because, whenever this habitat is present, it is often classified as not significantly representative of those sites, thus negating the need to evaluate the conservation degree in the SDFs.

In HD_ST, habitat type 2240 (“*Brachypodietalia* dune grasslands with annuals”) was found to have the highest percentage of conservation degree C (86%), with no records with a conservation degree A. This habitat is not priority one, but it is the equivalent of habitat 6220 “Pseudo-steppe with grasses and annuals of the *Thero-Brachypodietea*”, which is a priority habitat, in a dune context (Biondi et al. 2009). These features make it an alarming example that might suggest the need for immediate restoration efforts for habitats in such a critical state to avoid the risk of local extinction. However, Farris et al. (2007a) affirm that habitat 2240 has never been recorded in Sardinia, and that all Natura 2000 Forms records of this habitat should be attributed to habitat 2230 (“*Malcolmietalia* dune grasslands”), highlighting the lack of clarity of the Interpretation Manual of the 92/43/EEC Directive Habitat for some cases. This specific situation requires clarification, and thorough analysis is needed before planning any restoration process for these habitats.

Due to their low number, considerations for BD/HD_ST must necessarily be contextualized to individual sites. In addition to the lack of data for three sites, of the seven ones investigated, only three are almost entirely terrestrial (ITB020014 “Golfo di Orosei”, ITB021103 “Monti del Gennargentu”, ITB022212 “Supramonte di Oliena, Orgosolo e Urzulei - Su Sercone”). The other sites are completely marine areas (ITB030080 “Isola di Mal di Ventre e Catalano”) or islands with a preponderant percentage of marine area (ITB010008 “Arcipelago La Maddalena”, ITB040026 “Isola del Toro” and ITB040081 “Isola della Vacca”). The three terrestrial sites are located in a limited area of central-eastern Sardinia, characterized by the highest mountains of the island (Gennargentu) and distinctly calcareous areas (Supramonte), which strongly influence their vegetation (Fenu et al. 2010).

Although the lower number of sites reduces the statistical relevance, in this context, habitat 9340 (“*Quercus ilex* and *Quercus rotundifolia* forests”) has a conservation degree A in all three terrestrial sites, suggesting the good condition of forest ecosystems in these limited areas.

Habitat type 3170 (“Mediterranean temporary ponds”) has the most recorded habitats in conservation status B, as classified only with this rating. Habitat 3170 is a priority one, characterized by ephemeral vegetation linked to temporary ponds that disappear completely in the dry season (Zacharias and Zamparas 2010). The seasonality, high dependency on water fluctuations, and the localization in small areas make it difficult to evaluate the conservation status of this habitat (Poponessi et al. 2024), resulting in the absence of conservation degrees A and C. Due to their intrinsic characteristics, these habitats are difficult to conserve locally (Bagella and Caria 2013), making restoration measures equally complex and requiring in-depth, specific studies, also considering multi-taxa approaches (Cogoni et al. 2016).

### Contribution to NRR Targets: A Focus on HD_ST

Habitats of Habitat Directive Site Type (HD_ST) were considered to estimate the coverage of habitats that will undergo restoration measures and to assess the feasibility of NRR targets at the regional level.

While it was clear from the outset that the restoration target of 20% of the territory by 2030 could not be met solely by considering Natura 2000 areas, as they cover only 18.87% of the regional land, 3.7% is a good starting point. This is especially considering that habitats of Birds Directive Site Type and Birds Directive/Habitat Directive Site Type are not currently included, and that agricultural areas could also be subject to restoration efforts. Based on these results, reaching the 20% restoration target in Sardinia will necessarily require the implementation of restoration measures in agricultural and natural areas outside the existing Natura 2000 network.

It is assumed that defining the feasibility of achieving the NRR target of restoring 30% of degraded habitats will require a regional mapping of all habitats, both inside and outside Natura 2000 areas. However, considering the HD_ST sample, we can already see that the 30% target can be exceeded. Habitats outside Natura 2000 areas likely have a worse conservation degree than those within, as the latter are under regulations that protect them and are less affected by land use changes (Hermoso et al. 2018), making the goal feasible at this scale.

Since the 30% target can be largely achieved, this could suggest that restoration measures should initially focus on smaller and more threatened habitats to contribute first to achieving this target, while larger and more widespread habitats could then be restored to maximize the restored areas, focusing on the 20% target.

Of the ~893 km^2^ of habitats with a conservation degree B and C, around 80% consist of only seven habitat types: “Coastal lagoons” (HD code 1150), “Arborescent matorral with *Juniperus spp*.” (HD code 5210), “Thermo-Mediterranean and pre-desert scrub” (HD code 5330), “Pseudo-steppe with grasses and annuals of the *Thero-Brachypodietea*” (HD code 6220), “*Olea* and *Ceratonia* forests” (HD code 9320), “*Quercus suber* forests” (HD code 9330) and “*Quercus ilex* and *Quercus rotundifolia* forests” (HD code 9340). These habitats will therefore necessarily contribute the most to achieving the 20% target.

Habitat 1150, the only representative of the NRR Group 1 (“wetlands”), exhibits considerable ecological variability related to several factors such as salinity, water depth, temperature, substrate, and connectivity to the sea (Biondi et al. 2009). Restoration measures for this habitat, such as the transplantation of aquatic plants or improved water supply, require site-specific studies to propose appropriate solutions for improving the conservation degree (Bolpagni 2020; Bertolini and da Mosto 2021).

Habitat 6220 is a special case, as it can represent the early stages (pioneers) of colonization of new surfaces, such as rocky outcrops or degraded lands, as the main drivers of dry Mediterranean grasslands are grazing (Carmona et al. 2015) or repeated fire phenomena. The presence of this habitat is often linked to degradation processes and typically constitutes a pioneer stage that, in the absence of pressure, gives way to perennial shrub formations (Farris et al. 2007b). Thus, any restoration measures must be approached with caution. Restoration solutions for these habitats are particularly numerous, with specific strengths and weaknesses (Buisson et al. 2021).

Habitat types 5210 and 5330 are among those with the most recorded habitats with conservation degree A, but their extensive distribution also makes them the most represented among those with conservation degree B, which are valid for restoration. For regions with a thermo-Mediterranean bioclimate, like Sardinia, significant contributions to NRR targets can only be made by restoring these widely distributed habitats.

The physiognomy of Mediterranean scrub vegetation can vary enormously depending on the context. It ranges from the typical cenosis of rocky coastal areas to marked aridity of *Euphorbia dendroides*, from the dense garrigue of *Ampelodesmos mauritanicus* to the sparser with camephyte, or even matorral consisting of arborescent bushy species. Excluding contexts where environmental factors limit the development of potential natural vegetation (PNV) (sensu Farris et al. 2010), forest formations are expected to replace scrub and represent the final stage of ecological succession (Blasi et al. 2000; Amici et al. 2013). The restoration and preservation of the Mediterranean scrub will often lead to the development of tree formations in many contexts, which are more resilient to extreme events and serve as indicators of greater general naturality of the territory.

While restoring these habitats will undoubtedly improve ecological values, it is debatable whether focusing efforts on restoring large ecosystems to maximize the surface area of intervention is more effective than targeting smaller, more fragile ecosystems with high biodiversity value (Szangolies et al. 2022). Whether single large or several small patches benefit biodiversity remains a topic of debate (Fletcher et al. 2018; Fahrig et al. 2019).

Our study shows that, statistically, many small habitats in Sardinia, such as coastal habitats, have a poorer conservation status. These habitats have a high value for biodiversity and ecosystem services (Gleason et al. 2023), but due to their limited surface, their restoration would have minimal impact on achieving NRR targets. Conversely, only a few habitats are large enough to make a tangible contribution.

The NRR specifies that, regardless of reaching the target of restoring degraded ecosystems in 20% of the territory by 2030, all ecosystems in need of restoration must be restored by 2050. Therefore, every habitat will undergo restoration efforts; however, improper planning in the initial phases of the interventions could result in the local loss of some ecosystems, making them irrecoverable by the 2050 deadline or requiring significantly greater restoration efforts. Thorough planning at the local level is needed to avoid focusing solely on maximizing the area to be restored and overlooking habitats that are particularly at risk.

### Where to Intervene Fast? Localization of the Intervention

We observed that habitat dimensions are unevenly distributed across the island, with larger habitats found in the northern part. From this perspective, a management recommendation could involve conducting more detailed research in the southern area of the island to identify and restore smaller habitats with conservation degree C. For example, the sites ITB040051 “Bruncu de Su Monte Moru - Geremeas (Mari Pintau)”, ITB042218 “Stagno di Piscinnì” and ITB042231 “Tra Forte Village e Perla Marina” host habitats that match these characteristics and we, therefore, propose these sites for priority analysis.

Restoring the most endangered and smaller habitats would primarily contribute to achieving the 30% target. Restoration of habitats with conservation degree B could then take place in a second phase, focusing on the northern area of the island, where more extensive habitats are located. Among the sites with habitats matching these characteristics, we propose three sites for further investigation: ITB011102 “Catena del Marghine e del Goceano”, ITB020041 “Entroterra e zona costiera tra Bosa, Capo Marargiu e Porto Tangone” and ITB041111 “Monte Linas – Marganai”. Thus, the restoration of these habitats will provide the greatest contribution to achieving the 20% target.

Importantly, this regional-level analysis of Natura 2000 sites not only supports national planning under the NRR but also demonstrates a scalable and transferable methodology. By focusing on a finer territorial resolution, this research provides concrete, location-specific insights that can inform restoration strategies in other European regions with similar ecological and planning challenges. More broadly, it offers a replicable model for international contexts where habitat data are available at coarse resolution or where restoration priorities must be tailored to local conditions.

While a systematic survey of habitats across the entire island would yield more exhaustive results, this preparatory, qualitative analysis based on Natura 2000 Standard Data Forms already offers a clear indication of the ecosystems most in need of restoration and the spatial distribution of such needs. In particular, this approach highlights how regional-level insight can sharpen restoration planning and help translate continental or national targets into actionable strategies on the ground.

Given that the most up-to-date habitat cartography at the European level has a 10 km resolution, determining a region’s contribution to the NRR targets requires first creating a finer-resolution cartography of habitat conservation status, as expected by the NRR in preparation for national restoration plans. This study contributes to that objective as a starting point by proposing a replicable approach for generating spatially explicit, policy-relevant data that can enhance restoration planning both within and beyond Europe.

## Conclusion

This study demonstrates that Natura 2000 sites play a critical role in advancing the Nature Restoration Regulation at a regional scale, while also highlighting the limitations of relying solely on these areas to achieve ambitious restoration targets. By focusing on Sardinia, a region with high biodiversity and significant habitat variability, we provided a detailed assessment of habitat conservation degree and restoration potential.

The findings confirm that Natura 2000 sites can serve as a valuable starting point for restoration efforts, but achieving the NRR’s goals will require a broader, integrated approach. Restoration measures must extend beyond Natura 2000 to include agricultural, urban, and other semi-natural areas, particularly those with poorer conservation status.

The study also underscores the importance of prioritizing smaller, highly threatened habitats alongside larger, more extensive ecosystems. This dual approach can maximize biodiversity conservation while ensuring progress toward the NRR’s area-based targets.

Achieving the NRR’s long-term objectives will require systematic, high-resolution habitat mapping and the implementation of localized restoration plans across Europe. This work provides a replicable model for other regions, contributing to the foundational knowledge necessary to balance ecological, social, and economic priorities in restoration planning. By integrating these findings into broader national and European strategies, meaningful progress can be made toward restoring degraded ecosystems and safeguarding Europe’s natural heritage.

## Supplementary information

Supplementary information

## Data Availability

Data will be made available on request.
