# Supplementary material for: Are the Targets of the Nature Restoration Regulation Achievable at a Regional Scale? An Analysis of Natura 2000 Sites on the Island of Sardinia
Source: Environ Manage. 2025 Aug 22;75(12):3170–87. doi: 10.1007/s00267-025-02242-x (PMC12575458; doi:10.1007/s00267-025-02242-x)
Supplement: Supplementary file 1 — Supplementary information [file 267_2025_2242_MOESM1_ESM.docx]

### **Table S1**

List of terrestrial, coastal and freshwater habitat types and groups of habitat types as reported in Nature Restoration Regulation Annex I. The habitat types names and codes are referred to the list of Annex I of the Directive 92/43/EEC.

| NRR Group name | Habitat Directive code | Habitat Directive name |
| --- | --- | --- |
| Wetlands (coastal & inland) | 1130 | Estuaries |
| Wetlands (coastal & inland) | 1140 | Mudflats and sandflats not covered by seawater at low tide |
| Wetlands (coastal & inland) | 1150 | Coastal lagoons |
| Wetlands (coastal & inland) | 1310 | *Salicornia* and other annuals colonizing mud and sand |
| Wetlands (coastal & inland) | 1320 | Spartina swards (*Spartinion* *maritimae*) |
| Wetlands (coastal & inland) | 1330 | Atlantic salt meadows (*Glauco-Puccinellietalia maritimae*) |
| Wetlands (coastal & inland) | 1340 | Inland salt meadows |
| Wetlands (coastal & inland) | 1410 | Mediterranean salt meadows (*Juncetalia maritimi*) |
| Wetlands (coastal & inland) | 1420 | Mediterranean and thermo-Atlantic halophilous scrubs (*Sarcocornetea fruticosi*) |
| Wetlands (coastal & inland) | 1530 | Pannonic salt steppes and salt marshes |
| Wetlands (coastal & inland) | 1650 | Boreal Baltic narrow inlets |
| Wetlands (coastal & inland) | 4010 | Northern Atlantic wet heaths with *Erica tetralix* |
| Wetlands (coastal & inland) | 4020 | Temperate Atlantic wet heaths with *Erica ciliaris* and *Erica tetralix* |
| Wetlands (coastal & inland) | 6460 | Peat grasslands of Troodos |
| Wetlands (coastal & inland) | 7110 | Active raised bogs |
| Wetlands (coastal & inland) | 7120 | Degraded raised bogs still capable of natural regeneration |
| Wetlands (coastal & inland) | 7130 | Blanket bogs |
| Wetlands (coastal & inland) | 7140 | Transition mires and quaking bogs |
| Wetlands (coastal & inland) | 7150 | Depressions on peat substrates of the *Rhynchosporion* |
| Wetlands (coastal & inland) | 7160 | Fennoscandian mineral-rich springs and springfens |
| Wetlands (coastal & inland) | 7210 | Calcareous fens with *Cladium mariscus* and species of the *Caricion davallianae* |
| Wetlands (coastal & inland) | 7220 | Petrifying springs with tufa formation (*Cratoneurion*) |
| Wetlands (coastal & inland) | 7230 | Alkaline fens |
| Wetlands (coastal & inland) | 7240 | Alpine pioneer formations of the *Caricion bicoloris-atrofuscae* |
| Wetlands (coastal & inland) | 7310 | Aapa mires |
| Wetlands (coastal & inland) | 7320 | Palsa mires |
| Wetlands (coastal & inland) | 9080 | Fennoscandian deciduous swamp woods |
| Wetlands (coastal & inland) | 91D0 | Bog woodland |
| Grasslands and other pastoral habitats | 1630 | Boreal Baltic coastal meadows |
| Grasslands and other pastoral habitats | 21A0 | Machairs |
| Grasslands and other pastoral habitats | 4030 | European dry heaths |
| Grasslands and other pastoral habitats | 4040 | Dry Atlantic coastal heaths with *Erica vagans* |
| Grasslands and other pastoral habitats | 4090 | Endemic oro-Mediterranean heaths with gorse |
| Grasslands and other pastoral habitats | 5130 | *Juniperus communis* formations on heaths or calcareous grasslands |
| Grasslands and other pastoral habitats | 8240 | Limestone pavements |
| Grasslands and other pastoral habitats | 6110 | Rupicolous calcareous or basophilic grasslands of the *Alysso-Sedion albi* |
| Grasslands and other pastoral habitats | 6120 | Xeric sand calcareous grasslands |
| Grasslands and other pastoral habitats | 6130 | Calaminarian grasslands of the *Violetalia calaminariae* |
| Grasslands and other pastoral habitats | 6140 | Siliceous Pyrenean *Festuca eskia* grasslands |
| Grasslands and other pastoral habitats | 6150 | Siliceous alpine and boreal grasslands |
| Grasslands and other pastoral habitats | 6160 | Oro-Iberian *Festuca indigesta* grasslands |
| Grasslands and other pastoral habitats | 6170 | Alpine and subalpine calcareous grasslands |
| Grasslands and other pastoral habitats | 6180 | Macaronesian mesophile grasslands |
| Grasslands and other pastoral habitats | 6190 | Rupicolous pannonic grasslands (*Stipo-Festucetalia pallentis*) |
| Grasslands and other pastoral habitats | 6210 | Semi-natural dry grasslands and scrubland facies on calcareous substrates (*Festuco-Brometalia*) |
| Grasslands and other pastoral habitats | 6220 | Pseudo-steppe with grasses and annuals of the *Thero-Brachypodietea* |
| Grasslands and other pastoral habitats | 6230 | Species-rich Nardus grasslands, on silicious substrates in mountain areas (and submountain areas in Continental Europe) |
| Grasslands and other pastoral habitats | 6240 | Sub-Pannonic steppic grasslands |
| Grasslands and other pastoral habitats | 6250 | Pannonic loess steppic grasslands |
| Grasslands and other pastoral habitats | 6260 | Pannonic sand steppes |
| Grasslands and other pastoral habitats | 6270 | Fennoscandian lowland species-rich dry to mesic grasslands |
| Grasslands and other pastoral habitats | 6280 | Nordic alvar and precambrian calcareous flatrocks |
| Grasslands and other pastoral habitats | 62A0 | Eastern sub-Mediterranean dry grasslands (*Scorzoneratalia villosae*) |
| Grasslands and other pastoral habitats | 62B0 | Serpentinophilous grassland of Cyprus |
| Grasslands and other pastoral habitats | 62C0 | Ponto-Sarmatic steppes |
| Grasslands and other pastoral habitats | 62D0 | Oro-Moesian acidophilous grasslands |
| Grasslands and other pastoral habitats | 6410 | Molinia meadows on calcareous, peaty or clayey-silt-laden soils (*Molinion caeruleae*) |
| Grasslands and other pastoral habitats | 6420 | Mediterranean tall humid grasslands of the *Molinio-Holoschoenion* |
| Grasslands and other pastoral habitats | 6510 | Lowland hay meadows (*Alopecurus pratensis*, *Sanguisorba officinalis*) |
| Grasslands and other pastoral habitats | 6520 | Mountain hay meadows |
| Grasslands and other pastoral habitats | 6310 | Dehesas with evergreen *Quercus spp*. |
| Grasslands and other pastoral habitats | 6530 | Fennoscandian wooded meadows |
| Grasslands and other pastoral habitats | 9070 | Fennoscandian wooded pastures |
| River, lake, alluvial and riparian habitats | 3110 | Oligotrophic waters containing very few minerals of sandy plains (*Littorelletalia uniflorae*) |
| River, lake, alluvial and riparian habitats | 3120 | Oligotrophic waters containing very few minerals generally on sandy soils of the West Mediterranean, with *Isoetes spp*. |
| River, lake, alluvial and riparian habitats | 3130 | Oligotrophic to mesotrophic standing waters with vegetation of the *Littorelletea uniflorae* and/or of the *Isoëto-Nanojuncetea* |
| River, lake, alluvial and riparian habitats | 3140 | Hard oligo-mesotrophic waters with benthic vegetation of Chara spp. |
| River, lake, alluvial and riparian habitats | 3150 | Natural eutrophic lakes with *Magnopotamion* or *Hydrocharition* — type vegetation |
| River, lake, alluvial and riparian habitats | 3160 | Natural dystrophic lakes and ponds |
| River, lake, alluvial and riparian habitats | 3170 | Mediterranean temporary ponds |
| River, lake, alluvial and riparian habitats | 3180 | Turloughs |
| River, lake, alluvial and riparian habitats | 3190 | Lakes of gypsum karst |
| River, lake, alluvial and riparian habitats | 31A0 | Transylvanian hot-spring lotus beds |
| River, lake, alluvial and riparian habitats | 3210 | Fennoscandian natural rivers |
| River, lake, alluvial and riparian habitats | 3220 | Alpine rivers and the herbaceous vegetation along their banks |
| River, lake, alluvial and riparian habitats | 3230 | Alpine rivers and their ligneous vegetation with *Myricaria germanica* |
| River, lake, alluvial and riparian habitats | 3240 | Alpine rivers and their ligneous vegetation with *Salix elaeagnos* |
| River, lake, alluvial and riparian habitats | 3250 | Constantly flowing Mediterranean rivers with *Glaucium flavum* |
| River, lake, alluvial and riparian habitats | 3260 | Water courses of plain to montane levels with the *Ranunculion fluitantis* and *Callitricho-Batrachion* vegetation |
| River, lake, alluvial and riparian habitats | 3270 | Rivers with muddy banks with*Chenopodion rubri p.p.* and *Bidention p.p*. vegetation |
| River, lake, alluvial and riparian habitats | 3280 | Constantly flowing Mediterranean rivers with *Paspalo-Agrostidion* species and hanging curtains of *Salix* and *Populus alba* |
| River, lake, alluvial and riparian habitats | 3290 | Intermittently flowing Mediterranean rivers of the *Paspalo-Agrostidion* |
| River, lake, alluvial and riparian habitats | 32A0 | Tufa cascades of karstic rivers of the Dinaric Alps |
| River, lake, alluvial and riparian habitats | 6430 | Hydrophilous tall herb fringe communities of plains and of the montane to alpine levels |
| River, lake, alluvial and riparian habitats | 6440 | Alluvial meadows of river valleys of the *Cnidion dubii* |
| River, lake, alluvial and riparian habitats | 6450 | Northern boreal alluvial meadows |
| River, lake, alluvial and riparian habitats | 6540 | Sub-Mediterranean grasslands of the *Molinio-Hordeion secalini* |
| River, lake, alluvial and riparian habitats | 9160 | Sub-Atlantic and medio-European oak or oak-hornbeam forests of the *Carpinion betuli* |
| River, lake, alluvial and riparian habitats | 91E0 | Alluvial forests with *Alnus glutinosa* and *Fraxinus excelsior* (*Alno-Padion, Alnion incanae, Salicion albae*) |
| River, lake, alluvial and riparian habitats | 91F0 | Riparian mixed forests of *Quercus robur*, *Ulmus laevis* and *Ulmus minor*, *Fraxinus excelsior* or *Fraxinus angustifolia*, along the great rivers (*Ulmenion* *minoris*) |
| River, lake, alluvial and riparian habitats | 92A0 | *Salix alba* and *Populus alba* galleries |
| River, lake, alluvial and riparian habitats | 92B0 | Riparian formations on intermittent Mediterranean water courses with *Rhododendron ponticum*, *Salix* and others |
| River, lake, alluvial and riparian habitats | 92C0 | *Platanus orientalis* and *Liquidambar orientalis* woods (*Platanion orientalis*) |
| River, lake, alluvial and riparian habitats | 92D0 | Southern riparian galleries and thickets (*Nerio-Tamaricetea* and *Securinegion tinctoriae*) |
| River, lake, alluvial and riparian habitats | 9370 | Palm groves of Phoenix |
| Forests | 9010 | Western Taïga |
| Forests | 9020 | Fennoscandian hemiboreal natural old broad-leaved deciduous forests (*Quercus, Tilia, Acer, Fraxinus or Ulmus*) rich in epiphytes |
| Forests | 9030 | Natural forests of primary succession stages of landupheaval coast |
| Forests | 9040 | Nordic subalpine/subarctic forests with *Betula pubescens ssp. czerepanovii* |
| Forests | 9050 | Fennoscandian herb-rich forests with *Picea abies* |
| Forests | 9060 | Coniferous forests on, or connected to, glaciofluvial eskers |
| Forests | 9110 | Luzulo-Fagetum beech forests |
| Forests | 9120 | Atlantic acidophilous beech forests with *Ilex* and sometimes also *Taxus* in the shrub layer (*Quercion robori-petraeae* or *Ilici-Fagenion*) |
| Forests | 9130 | *Asperulo-Fagetum* beech forests |
| Forests | 9140 | Medio-European subalpine beech woods with *Acer* and *Rumex arifolius* |
| Forests | 9150 | Medio-European limestone beech forests of the *Cephalanthero-Fagion* |
| Forests | 9170 | *Galio-Carpinetum* oak-hornbeam forests |
| Forests | 9180 | *Tilio-Acerion* forests of slopes, screes and ravines |
| Forests | 9190 | Old acidophilous oak woods with *Quercus robur* on sandy plains |
| Forests | 91A0 | Old sessile oak woods with *Ilex* and *Blechnum* in the British Isles |
| Forests | 91B0 | Thermophilous *Fraxinus angustifolia* woods |
| Forests | 91G0 | Pannonic woods with *Quercus petraea* and *Carpinus betulus* |
| Forests | 91H0 | Pannonian woods with *Quercus pubescens* |
| Forests | 91I0 | Euro-Siberian steppic woods with *Quercus spp*. |
| Forests | 91J0 | *Taxus baccata* woods of the British Isles |
| Forests | 91K0 | Illyrian *Fagus sylvatica* forests (*Aremonio-Fagion*) |
| Forests | 91L0 | Illyrian oak-hornbeam forests (*Erythronio-Carpinion*) |
| Forests | 91M0 | Pannonian-Balkanic turkey oak – sessile oak forests |
| Forests | 91P0 | Holy Cross fir forest (*Abietetum polonicum*) |
| Forests | 91Q0 | Western Carpathian calcicolous *Pinus sylvestris*forests |
| Forests | 91R0 | Dinaric dolomite Scots pine forests (*Genisto januensis-Pinetum*) |
| Forests | 91S0 | Western Pontic beech forests |
| Forests | 91T0 | Central European lichen Scots pine forests |
| Forests | 91U0 | Sarmatic steppe pine forest |
| Forests | 91V0 | Dacian Beech forests (*Symphyto-Fagion*) |
| Forests | 91W0 | Moesian beech forests |
| Forests | 91X0 | Dobrogean beech forests |
| Forests | 91Y0 | Dacian oak & hornbeam forests |
| Forests | 91Z0 | Moesian silver lime woods |
| Forests | 91AA | Eastern white oak woods |
| Forests | 91BA | Moesian silver fir forests |
| Forests | 91CA | Rhodopide and Balkan Range Scots pine forests |
| Forests | 9210 | Apeninne beech forests with *Taxus* and *Ilex* |
| Forests | 9220 | Apennine beech forests with *Abies alba* and beech forests with *Abies nebrodensis* |
| Forests | 9230 | Galicio-Portuguese oak woods with *Quercus robur* and *Quercus pyrenaica* |
| Forests | 9240 | *Quercus faginea* and Qu*ercus canariensis* Iberian woods |
| Forests | 9250 | *Quercus trojana* woods |
| Forests | 9260 | *Castanea sativa* woods |
| Forests | 9270 | Hellenic beech forests with *Abies borisii-regis* |
| Forests | 9280 | *Quercus frainetto* woods |
| Forests | 9290 | Cupressus forests (*Acero-Cupression*) |
| Forests | 9310 | Aegean *Quercus brachyphylla* woods |
| Forests | 9320 | *Olea* and *Ceratonia* forests |
| Forests | 9330 | *Quercus suber* forests |
| Forests | 9340 | *Quercus ilex* and *Quercus rotundifolia* forests |
| Forests | 9350 | *Quercus macrolepis* forests |
| Forests | 9360 | Macaronesian laurel forests (*Laurus, Ocotea*) |
| Forests | 9380 | Forests of *Ilex aquifolium* |
| Forests | 9390 | Scrub and low forest vegetation with *Quercus alnifolia* |
| Forests | 93A0 | Woodlands with *Quercus infectoria* (*Anagyro foetidae-Quercetum infectoriae*) |
| Forests | 9410 | Acidophilous *Picea* forests of the montane to alpine levels (*Vaccinio-Piceetea*) |
| Forests | 9420 | Alpine *Larix decidua* and/or *Pinus cembra* forests |
| Forests | 9430 | Subalpine and montane *Pinus uncinata* forests |
| Forests | 9510 | Southern Apennine *Abies alba* forests |
| Forests | 9520 | *Abies pinsapo* forests |
| Forests | 9530 | (Sub-) Mediterranean pine forests with endemic black pines |
| Forests | 9540 | Mediterranean pine forests with endemic Mesogean pines |
| Forests | 9550 | Canarian endemic pine forests |
| Forests | 9560 | Endemic forests with *Juniperus spp.* |
| Forests | 9570 | Tetraclinis articulata forests |
| Forests | 9580 | Mediterranean *Taxus baccata* woods |
| Forests | 9590 | *Cedrus brevifolia* forests (*Cedrosetum brevifoliae*) |
| Forests | 95A0 | High oro-Mediterranean pine forests |
| Steppe, heath and scrub habitats | 1430 | Halo-nitrophilous scrubs (*Pegano-Salsoletea*) |
| Steppe, heath and scrub habitats | 1510 | Mediterranean salt steppes (*Limonietalia*) |
| Steppe, heath and scrub habitats | 1520 | Iberian gypsum vegetation (*Gypsophiletalia*) |
| Steppe, heath and scrub habitats | 4050 | Endemic macaronesian heaths |
| Steppe, heath and scrub habitats | 4060 | Alpine and Boreal heaths |
| Steppe, heath and scrub habitats | 4070 | Bushes with *Pinus mugo* and *Rhododendron hirsutum*(*Mugo-Rhododendretum hirsuti*) |
| Steppe, heath and scrub habitats | 4080 | Sub-Arctic *Salix spp.* scrub |
| Steppe, heath and scrub habitats | 40A0 | Subcontinental peri-Pannonic scrub |
| Steppe, heath and scrub habitats | 40B0 | Rhodope *Potentilla fruticosa* thickets |
| Steppe, heath and scrub habitats | 40C0 | Ponto-Sarmatic deciduous thickets |
| Steppe, heath and scrub habitats | 5110 | Stable xerothermophilous formations with *Buxus sempervirens* on rock slopes (*Berberidion p.p*.) |
| Steppe, heath and scrub habitats | 5120 | Mountain *Cytisus purgans* formations |
| Steppe, heath and scrub habitats | 5140 | *Cistus palhinhae* formations on maritime wet heaths |
| Steppe, heath and scrub habitats | 5210 | Arborescent matorral with *Juniperus spp.* |
| Steppe, heath and scrub habitats | 5220 | Arborescent matorral with*Zyziphus* |
| Steppe, heath and scrub habitats | 5230 | Arborescent matorral with *Laurus nobilis* |
| Steppe, heath and scrub habitats | 5310 | *Laurus nobilis* thickets |
| Steppe, heath and scrub habitats | 5320 | Low formations of *Euphorbia* close to cliffs |
| Steppe, heath and scrub habitats | 5330 | Thermo-Mediterranean and pre-desert scrub |
| Steppe, heath and scrub habitats | 5410 | West Mediterranean clifftop phryganas (*Astragalo-Plantaginetum subulatae*) |
| Steppe, heath and scrub habitats | 5420 | *Sarcopoterium spinosum* phryganas |
| Steppe, heath and scrub habitats | 5430 | Endemic phryganas of the *Euphorbio-Verbascion* |
| Rocky and dune habitats | 1210 | Annual vegetation of drift lines |
| Rocky and dune habitats | 1220 | Perennial vegetation of stony banks |
| Rocky and dune habitats | 1230 | Vegetated sea cliffs of the Atlantic and Baltic Coasts |
| Rocky and dune habitats | 1240 | Vegetated sea cliffs of the Mediterranean coasts with endemic *Limonium spp.* |
| Rocky and dune habitats | 1250 | Vegetated sea cliffs with endemic flora of the Macaronesian coasts |
| Rocky and dune habitats | 1610 | Baltic esker islands with sandy, rocky and shingle beach vegetation and sublittoral vegetation |
| Rocky and dune habitats | 1620 | Boreal Baltic islets and small islands |
| Rocky and dune habitats | 1640 | Boreal Baltic sandy beaches with perennial vegetation |
| Rocky and dune habitats | 2110 | Embryonic shifting dunes |
| Rocky and dune habitats | 2120 | Shifting dunes along the shoreline with *Ammophila arenaria* (‘white dunes’) |
| Rocky and dune habitats | 2130 | Fixed coastal dunes with herbaceous vegetation (‘grey dunes’) |
| Rocky and dune habitats | 2140 | Decalcified fixed dunes with *Empetrum nigrum* |
| Rocky and dune habitats | 2150 | Atlantic decalcified fixed dunes (*Calluno-Ulicetea*) |
| Rocky and dune habitats | 2160 | Dunes with *Hippophaë rhamnoides* |
| Rocky and dune habitats | 2170 | Dunes with *Salix repens ssp. argentea* (*Salicion arenariae*) |
| Rocky and dune habitats | 2180 | Wooded dunes of the Atlantic, Continental and Boreal region |
| Rocky and dune habitats | 2190 | Humid dune slacks |
| Rocky and dune habitats | 2210 | *Crucianellion maritimae* fixed beach dunes |
| Rocky and dune habitats | 2220 | Dunes with *Euphorbia terracina* |
| Rocky and dune habitats | 2230 | *Malcolmietalia* dune grasslands |
| Rocky and dune habitats | 2240 | *Brachypodietalia* dune grasslands with annuals |
| Rocky and dune habitats | 2250 | Coastal dunes with *Juniperus spp.* |
| Rocky and dune habitats | 2260 | *Cisto-Lavenduletalia* dune sclerophyllous scrubs |
| Rocky and dune habitats | 2270 | Wooded dunes with *Pinus pinea* and/or P*inus pinaster* |
| Rocky and dune habitats | 2310 | Dry sand heaths with *Calluna* and *Genista* |
| Rocky and dune habitats | 2320 | Dry sand heaths with Calluna and Empetrum nigrum |
| Rocky and dune habitats | 2330 | Inland dunes with open *Corynephorus* and *Agrostis* grasslands |
| Rocky and dune habitats | 2340 | Pannonic inland dunes |
| Rocky and dune habitats | 91N0 | Pannonic inland sand dune thicket (*Junipero-Populetum albae*) |
| Rocky and dune habitats | 8110 | Siliceous scree of the montane to snow levels (*Androsacetalia alpinae* and *Galeopsietalia ladani*) |
| Rocky and dune habitats | 8120 | Calcareous and calcshist screes of the montane to alpine levels (*Thlaspietea rotundifolii*) |
| Rocky and dune habitats | 8130 | Western Mediterranean and thermophilous scree |
| Rocky and dune habitats | 8140 | Eastern Mediterranean screes |
| Rocky and dune habitats | 8150 | Medio-European upland siliceous screes |
| Rocky and dune habitats | 8160 | Medio-European calcareous scree of hill and montane levels |
| Rocky and dune habitats | 8210 | Calcareous rocky slopes with chasmophytic vegetation |
| Rocky and dune habitats | 8220 | Siliceous rocky slopes with chasmophytic vegetation |
| Rocky and dune habitats | 8230 | Siliceous rock with pioneer vegetation of the *Sedo-Scleranthion* or of the*Sedo albi-Veronicion dillenii* |
| Rocky and dune habitats | 8310 | Caves not open to the public |
| Rocky and dune habitats | 8320 | Fields of lava and natural excavations |
| Rocky and dune habitats | 8340 | Permanent glaciers |

### **Table S2**

List of habitat types with the habitat records per different degrees of conservation in the Natura 2000 network. The highest value for the respective degree of conservation is highlighted in bold.

|  | Degree of conservation | | | |
| --- | --- | --- | --- | --- |
| Habitat Directive code | 0 | A | B | C |
| 1130 | 1 | 1 | 1 |  |
| 1150 | 5 | 16 | 23 | 5 |
| 1210 | 4 | 8 | 19 | **25** |
| 1240 | 5 | **35** | 11 | 3 |
| 1310 | 8 | 9 | 11 | 2 |
| 1320 | 1 |  |  |  |
| 1410 | 4 | 10 | 25 | 14 |
| 1420 | 2 | 12 | 26 | 12 |
| 1430 | 5 |  | 1 | 5 |
| 1510 | 4 | 12 | 20 | 3 |
| 2110 | 7 | 2 | 19 | 24 |
| 2120 | **9** | 4 | 13 | 16 |
| 2210 | 5 | 5 | 18 | 19 |
| 2230 | 5 | 5 | 26 | 9 |
| 2240 | 1 |  | 2 | 20 |
| 2250 | 4 | 5 | 22 | 10 |
| 2260 | 4 |  | 9 | 1 |
| 2270 | 3 | 1 | 13 | 8 |
| 3120 | 1 |  | 10 | 2 |
| 3130 | 1 | 2 | 14 | 1 |
| 3140 |  | 1 | 2 |  |
| 3150 | 3 |  | 1 | 2 |
| 3170 | 4 | 5 | 14 | 2 |
| 3250 |  |  |  | 1 |
| 3260 |  |  | 2 |  |
| 3280 | 7 |  | 1 | 1 |
| 3290 |  | 3 | 1 | 1 |
| 4060 |  |  | 1 |  |
| 4090 |  | 1 | 4 |  |
| 5130 | 1 |  |  |  |
| 5210 | 4 | 12 | 33 | 5 |
| 5230 |  | 4 | 5 | 1 |
| 5320 | 1 | 19 | 3 | 1 |
| 5330 | 5 | 20 | **40** | 11 |
| 5410 |  | 6 | 1 | 1 |
| 5430 | 2 | 19 | 14 | 2 |
| 6210 |  |  | 1 |  |
| 6220 | **9** | 14 | 31 | 17 |
| 6310 | 2 |  | 12 | 3 |
| 6420 | 1 | 1 | 4 | 2 |
| 7220 |  | 1 |  |  |
| 8130 |  |  | 3 | 2 |
| 8210 |  | 10 | 3 |  |
| 8220 | 2 | 1 | 4 |  |
| 8310 | 2 | 10 | 3 | 2 |
| 9260 |  |  |  | 1 |
| 9320 | 3 | 8 | 26 | 6 |
| 9330 | 4 | 3 | 16 | 2 |
| 9340 | 8 | 13 | 24 | 10 |
| 9380 | 1 | 2 | 3 |  |
| 9540 | 1 | 2 | 3 | 2 |
| 9560 |  |  | 1 |  |
| 9580 | 1 | 7 | 2 |  |
| 91AA | 7 |  |  |  |
| 91E0 | 5 | 5 | 5 | 2 |
| 92A0 | 3 | 1 | 8 | 4 |
| 92D0 | **9** | 12 | 21 | 15 |

### **Table S3**

List of habitat types with the habitat records per different degree of conservation in the BD_ST. The highest value for the respective degree of conservation is highlighted in bold.

|  | Degree of conservation | | | |
| --- | --- | --- | --- | --- |
| Habitat Directive code | 0 | A | B | C |
| 1130 |  | 1 | 1 |  |
| 1150 | 3 | 6 | 4 |  |
| 1210 | 3 | 1 | 2 | 5 |
| 1240 | 2 | **7** | 1 | 2 |
| 1310 | **6** | 1 | 1 | 1 |
| 1320 | 1 |  |  |  |
| 1410 | 3 | 3 | 3 | **7** |
| 1420 | 2 | 3 | 5 | **7** |
| 1430 | 1 |  |  | 2 |
| 1510 | 2 | 2 | 6 | 1 |
| 2110 | 2 |  | 1 | 3 |
| 2120 | 2 |  | 3 | 1 |
| 2210 | 3 |  | 1 | 1 |
| 2230 | 4 | 1 | 2 |  |
| 2250 | 1 | 1 | 2 | 2 |
| 2270 | 1 |  | 1 | 2 |
| 3120 |  |  | 2 |  |
| 3130 | 1 |  | 5 |  |
| 3150 | 1 |  |  | 1 |
| 3170 | 4 | 2 | 3 |  |
| 3260 |  |  | 1 |  |
| 3280 | 3 |  |  |  |
| 5210 | 2 | 4 | 5 | 2 |
| 5230 |  | 1 | 2 | 1 |
| 5320 | 1 | 5 |  |  |
| 5330 |  | 3 | **10** | 4 |
| 5410 |  | 2 |  |  |
| 5430 |  | 4 | 5 | 1 |
| 6210 |  |  | 1 |  |
| 6220 | 4 | 4 | 7 | 5 |
| 6310 | 1 |  | 4 |  |
| 6420 | 1 | 1 | 1 | 1 |
| 8130 |  |  | 1 |  |
| 8210 |  | 2 |  |  |
| 8310 | 1 | 1 | 1 |  |
| 9320 | 2 | 1 | 7 | 1 |
| 9330 | 1 |  | 6 |  |
| 9340 | 2 | 1 | 9 | 2 |
| 9540 |  | 1 | 1 | 1 |
| 9580 |  | 2 |  |  |
| 91AA | 1 |  |  |  |
| 91E0 |  | 2 |  |  |
| 92A0 | 1 |  | 1 |  |
| 92D0 | 5 | 1 | 3 | 4 |

### **Table S4**

List of habitat types with the habitat records per different degree of conservation in the HD_ST. The highest value for the respective degree of conservation is highlighted in bold.

|  | Degree of conservation | | | |
| --- | --- | --- | --- | --- |
| Habitat Directive code | 0 | A | B | C |
| 1130 | 1 |  |  |  |
| 1150 | 2 | 10 | 18 | 5 |
| 1210 | 1 | 7 | 16 | 18 |
| 1240 | 2 | **26** | 10 | 1 |
| 1310 | 2 | 8 | 8 | 1 |
| 1410 | 1 | 7 | 20 | 7 |
| 1420 |  | 9 | 19 | 5 |
| 1430 | 3 |  | 1 | 3 |
| 1510 | 1 | 10 | 14 | 2 |
| 2110 | 4 | 2 | 18 | **19** |
| 2120 | 5 | 4 | 9 | 15 |
| 2210 | 1 | 5 | 17 | 17 |
| 2230 | 1 | 4 | 23 | 9 |
| 2240 | 1 |  | 2 | **19** |
| 2250 | 3 | 4 | 19 | 8 |
| 2260 | 4 |  | 9 | 1 |
| 2270 | 2 | 1 | 12 | 6 |
| 3120 | 1 |  | 5 | 2 |
| 3130 |  | 2 | 6 | 1 |
| 3140 |  | 1 | 1 |  |
| 3150 | 2 |  | 1 | 1 |
| 3170 |  | 3 | 7 | 2 |
| 3260 |  |  | 1 |  |
| 3280 | 4 |  | 1 | 1 |
| 3290 |  | 2 | 1 | 1 |
| 4090 |  | 1 | 3 |  |
| 5210 | 2 | 6 | 26 | 3 |
| 5230 |  | 3 | 3 |  |
| 5320 |  | 12 | 3 | 1 |
| 5330 | 5 | 15 | **28** | 7 |
| 5410 |  | 4 | 1 | 1 |
| 5430 | 2 | 13 | 8 | 1 |
| 6220 | 4 | 9 | 22 | 11 |
| 6310 | 1 |  | 7 | 3 |
| 6420 |  |  | 3 | 1 |
| 8130 |  |  | 1 | 1 |
| 8210 |  | 6 | 3 |  |
| 8220 | 2 | 1 | 3 |  |
| 8310 | 1 | 6 | 2 | 2 |
| 9260 |  |  |  | 1 |
| 9320 | 1 | 6 | 17 | 5 |
| 9330 | 3 | 3 | 10 | 2 |
| 9340 | **6** | 9 | 15 | 7 |
| 9380 |  | 2 | 2 |  |
| 9540 | 1 | 1 | 1 | 1 |
| 9580 |  | 4 | 2 |  |
| 91AA | 5 |  |  |  |
| 91E0 | 5 | 2 | 4 | 1 |
| 92A0 | 2 | 1 | 7 | 3 |
| 92D0 | 3 | 9 | 18 | 10 |

### **Table S5**

List of habitat types with the habitat records per different degree of conservation in the BD/HD_ST. The highest value for the respective degree of conservation is highlighted in bold.

|  | Degree of conservation | | | |
| --- | --- | --- | --- | --- |
| Habitat Directive code | 0 | A | B | C |
| 1150 |  |  | 1 |  |
| 1210 |  |  | 1 | **2** |
| 1240 | 1 | 2 |  |  |
| 1310 |  |  | 2 |  |
| 1410 |  |  | 2 |  |
| 1420 |  |  | 2 |  |
| 1430 | 1 |  |  |  |
| 1510 | 1 |  |  |  |
| 2110 | 1 |  |  | **2** |
| 2120 | **2** |  | 1 |  |
| 2210 | 1 |  |  | 1 |
| 2230 |  |  | 1 |  |
| 2240 |  |  |  | 1 |
| 2250 |  |  | 1 |  |
| 3120 |  |  | 3 |  |
| 3130 |  |  | 3 |  |
| 3140 |  |  | 1 |  |
| 3170 |  |  | **4** |  |
| 3250 |  |  |  | 1 |
| 3290 |  | 1 |  |  |
| 4060 |  |  | 1 |  |
| 4090 |  |  | 1 |  |
| 5130 | 1 |  |  |  |
| 5210 |  | 2 | 2 |  |
| 5320 |  | 2 |  |  |
| 5330 |  | 2 | 2 |  |
| 5430 |  | 2 | 1 |  |
| 6220 | 1 | 1 | 2 | 1 |
| 6310 |  |  | 1 |  |
| 7220 |  | 1 |  |  |
| 8130 |  |  | 1 | 1 |
| 8210 |  | 2 |  |  |
| 8220 |  |  | 1 |  |
| 8310 |  | **3** |  |  |
| 9320 |  | 1 | 2 |  |
| 9340 |  | **3** |  | 1 |
| 9380 | 1 |  | 1 |  |
| 9540 |  |  | 1 |  |
| 9560 |  |  | 1 |  |
| 9580 | 1 | 1 |  |  |
| 91AA | 1 |  |  |  |
| 91E0 |  | 1 | 1 | 1 |
| 92A0 |  |  |  | 1 |
| 92D0 | 1 | 2 |  | 1 |

### **Table S6**

Surface coverage (ha) of the NRR groups per degree of conservation in BD_ST.

|  | Surface cover (ha) per degree of conservation | | |  |
| --- | --- | --- | --- | --- |
| NRR Group | 0 | A | B | C |
| Wetlands (coastal & inland) | 800,37 | 3993,29 | 2974,38 | 578,19 |
| Grasslands and other pastoral habitats | 219,09 | 4957,26 | 12181,86 | 2031,07 |
| River, lake, alluvial and riparian habitats | 1521,34 | 263,91 | 922,58 | 189,65 |
| Forests | 424,12 | 1467,86 | 19014,75 | 427,99 |
| Steppe, heath and scrub habitats | 130,66 | 5636,71 | 3349,61 | 772,82 |
| Rocky and dune habitats | 238,37 | 1216,69 | 888,14 | 464,69 |

### **Table S7**

Surface coverage (ha) of the NRR groups per degree of conservation in HD_ST.

|  | Surface cover (ha) per degree of conservation | | | |
| --- | --- | --- | --- | --- |
| NRR Group | 0 | A | B | C |
| Wetlands (coastal & inland) | 75,45 | 4336,68 | 6671,96 | 804,02 |
| Grasslands and other pastoral habitats | 2488,87 | 1867,74 | 11979,82 | 3334,46 |
| River, lake, alluvial and riparian habitats | 760,92 | 517,32 | 1117,41 | 1704,41 |
| Forests | 1316,83 | 38671,71 | 22561,72 | 5702,86 |
| Steppe, heath and scrub habitats | 850,72 | 8972,58 | 28689,01 | 1389,04 |
| Rocky and dune habitats | 94,27 | 2029,51 | 3283,00 | 2060,52 |

### **Table S8**

Surface coverage (ha) of the NRR groups per degree of conservation in BD/HD_ST.

|  | Surface cover (ha) per degree of conservation | | | |
| --- | --- | --- | --- | --- |
| NRR Group | 0 | A | B | C |
| Wetlands (coastal & inland) | | 1,21 | 29,24 |  |
| Grasslands and other pastoral habitats | 3,60 | 1078,90 | 4783,24 | 245,45 |
| River, lake, alluvial and riparian habitats | 4,43 | 241,42 | 23,35 | 77,02 |
| Forests | 58,03 | 21703,43 | 1425,50 | 12,08 |
| Steppe, heath and scrub habitats | 10,20 | 12849,18 | 3278,16 |  |
| Rocky and dune habitats | 3,17 | 2755,33 | 40,65 | 6,13 |

### **Table S9**

One-way ANOVA (non parametric) test (Kruskal-Wallis test) results for habitat surface coverage between the 3 degrees of conservation. In bold are highlighted the values that show significant differences.

|  | p value | Kruskal-Wallis |
| --- | --- | --- |
| General | **<0,0001** | 28,16 |
| Wetlands (coastal & inland) | 0,3971 | 1,847 |
| Grasslands and other pastoral habitats | 0,5024 | 1,377 |
| River, lake, alluvial and riparian habitats | 0,9561 | 0,08979 |
| Forests | 0,5269 | 1,282 |
| Steppe, heath and scrub habitats | 0,6342 | 0,9107 |
| Rocky and dune habitats | **0,0027** | 11,82 |

### **Table S10**

Unpaired T-test (non parametric) (Mann-Whitney test) results for habitat surface coverage between the degree of conservation A and the degree of conservation B-C together (habitats in need of restoration). In bold are highlighted the values that show significant differences.

|  | p value | Mann-Whitney U |
| --- | --- | --- |
| General | **0,0084** | 59097 |
| Wetlands (coastal & inland) | 0,35 | 1255 |
| Grasslands and other pastoral habitats | 0,34 | 201 |
| River, lake, alluvial and riparian habitats | 0,8528 | 719,5 |
| Forests | 0,8849 | 771,5 |
| Steppe, heath and scrub habitats | 0,6925 | 3095 |
| Rocky and dune habitats | **0,0134** | 6334 |

### **Table S11a**

One-way ANOVA (non parametric) test (Kruskal-Wallis test) results for habitat surface coverage and geographic location parameters (longitude and latitude) between the 3 degrees of conservation. In bold are highlighted the values that show significant differences.

| Kruskal-Wallis | | | | | | | |
| --- | --- | --- | --- | --- | --- | --- | --- |
|  | | χ² | | gdl | | p | |
| Surface |  | 28.160 |  | 2 |  | **< .001** |  |
| Longitude |  | 0.961 |  | 2 |  | 0.618 |  |
| Latitude |  | 15.697 |  | 2 |  | **< .001** |  |
|  | | | | | | | |

### **Table S11b**

Dwass-Steel-Critchlow-Fligner test results for habitat surface coverage and geographic location parameters (longitude and latitude), a pairwise comparison between the degrees of conservation. In bold are highlighted the values that show significant differences.

| Pairwise comparisons – Surface | | | |  | Pairwise comparisons - Longitude | | | |  | Pairwise comparisons - Latitude | | | |
| --- | --- | --- | --- | --- | --- | --- | --- | --- | --- | --- | --- | --- | --- |
|  |  | **W** | **p** |  |  |  | **W** | **p** |  |  |  | **W** | **p** |
| A | B | -1.38 | 0.595 |  | A | B | 1.202 | 0.672 |  | A | B | -0.439 | 0.948 |
| A | C | -6.85 | **< .001** |  | A | C | 1.143 | 0.698 |  | A | C | -4.629 | **0.003** |
| B | C | -6.51 | **< .001** |  | B | C | 0.528 | 0.926 |  | B | C | -5.261 | **< .001** |

### **Table S12a**

One-way ANOVA (non parametric) test (Kruskal-Wallis test) results for habitat surface coverage and geographic location parameters (longitude and latitude) between the 3 degrees of conservation for NRR group Wetlands (coastal & inland). In bold are highlighted the values that show significant differences.

| Kruskal-Wallis | | | | | | | |
| --- | --- | --- | --- | --- | --- | --- | --- |
|  | | **χ²** | | **df** | | **p** | |
| Surface |  | 1.85 |  | 2 |  | 0.397 |  |
| Longitude |  | 3.01 |  | 2 |  | 0.222 |  |
| Latitude |  | 14.97 |  | 2 |  | **< .001** |  |
|  | | | | | | | |

### **Table S12b**

Dwass-Steel-Critchlow-Fligner test results for habitat surface coverage and geographic location parameters (longitude and latitude), a pairwise comparison between the degrees of conservation in NRR group Wetlands (coastal & inland). In bold are highlighted the values that show significant differences.

| Pairwise comparisons – Surface | | | |  | Pairwise comparisons - Longitude | | | |  | Pairwise comparisons – Latitude | | | |
| --- | --- | --- | --- | --- | --- | --- | --- | --- | --- | --- | --- | --- | --- |
|  |  | **W** | **p** |  |  |  | **W** | **p** |  |  |  | **W** | **p** |
| A | B | -0.891 | 0.804 |  | A | B | -1.470 | 0.552 |  | A | B | 1.19 | 0.678 |
| A | C | -1.931 | 0.359 |  | A | C | 0.531 | 0.925 |  | A | C | -4.32 | **0.006** |
| B | C | -1.375 | 0.595 |  | B | C | 2.416 | 0.202 |  | B | C | -5.35 | **< .001** |

### **Table S13a**

One-way ANOVA (non parametric) test (Kruskal-Wallis test) results for habitat surface coverage and geographic location parameters (longitude and latitude) between the 3 degrees of conservation for NRR group Grassland and other pastoral habitats.

| Kruskal-Wallis | | | | | | | |
| --- | --- | --- | --- | --- | --- | --- | --- |
|  | | **χ²** | | **df** | | **p** | |
| Surface |  | 1.377 |  | 2 |  | 0.502 |  |
| Longitude |  | 1.208 |  | 2 |  | 0.547 |  |
| Latitude |  | 0.121 |  | 2 |  | 0.941 |  |
|  | | | | | | | |

### **Table S13b**

Dwass-Steel-Critchlow-Fligner test results for habitat surface coverage and geographic location parameters (longitude and latitude), a pairwise comparison between the degrees of conservation in NRR group Grassland and other pastoral habitats.

| Pairwise comparisons - Surface | | | |  | Pairwise comparisons - Longitude | | | |  | Pairwise comparisons - Latitude | | | | |
| --- | --- | --- | --- | --- | --- | --- | --- | --- | --- | --- | --- | --- | --- | --- |
|  |  | **W** | **p** |  |  |  | **W** | **p** |  |  |  | **W** | **p** |  |
| A | B | 1.660 | 0.469 |  | A | B | 1.429 | 0.570 |  | A | B | 0.3863 | 0.960 |  |
| A | C | 0.471 | 0.941 |  | A | C | 1.335 | 0.613 |  | A | C | 0.5497 | 0.920 |  |
| B | C | -0.853 | 0.819 |  | B | C | 0.390 | 0.959 |  | B | C | 0.0899 | 0.998 |  |

### **Table S14a**

One-way ANOVA (non parametric) test (Kruskal-Wallis test) results for habitat surface coverage and geographic location parameters (longitude and latitude) between the 3 degrees of conservation for NRR group River, lake, alluvial and riparian habitats. In bold are highlighted the values that show significant differences.

| Kruskal-Wallis | | | | | | | |
| --- | --- | --- | --- | --- | --- | --- | --- |
|  | | **χ²** | | **df** | | **p** | |
| Surface |  | 0.0898 |  | 2 |  | 0.956 |  |
| Longitude |  | 2.4877 |  | 2 |  | 0.288 |  |
| Latitude |  | 10.4558 |  | 2 |  | **0.005** |  |
|  | | | | | | | |

### **Table S14b**

Dwass-Steel-Critchlow-Fligner test results for habitat surface coverage and geographic location parameters (longitude and latitude), a pairwise comparison between the degrees of conservation in NRR group River, lake, alluvial and riparian habitats. In bold are highlighted the values that show significant differences.

| Pairwise comparisons - Surface | | | |  | Pairwise comparisons - Longitude | | | |  | Pairwise comparisons - Latitude | | | |
| --- | --- | --- | --- | --- | --- | --- | --- | --- | --- | --- | --- | --- | --- |
|  |  | **W** | **p** |  |  |  | **W** | **p** |  |  |  | **W** | **p** |
| A | B | 0.133 | 0.995 |  | A | B | -2.056 | 0.314 |  | A | B | 1.43 | 0.569 |
| A | C | 0.463 | 0.943 |  | A | C | -1.747 | 0.432 |  | A | C | -1.85 | 0.389 |
| B | C | 0.301 | 0.975 |  | B | C | 0.753 | 0.856 |  | B | C | -4.77 | **0.002** |

### **Table S15a**

One-way ANOVA (non parametric) test (Kruskal-Wallis test) results for habitat surface coverage and geographic location parameters (longitude and latitude) between the 3 degrees of conservation for NRR group Forests.

| Kruskal-Wallis | | | | | | | |
| --- | --- | --- | --- | --- | --- | --- | --- |
|  | | **χ²** | | **df** | | **p** | |
| Surface |  | 1.28 |  | 2 |  | 0.527 |  |
| Longitude |  | 5.49 |  | 2 |  | 0.064 |  |
| Latitude |  | 3.23 |  | 2 |  | 0.199 |  |
|  | | | | | | | |

### **Table S15b**

Dwass-Steel-Critchlow-Fligner test results for habitat surface coverage and geographic location parameters (longitude and latitude), a pairwise comparison between the degrees of conservation in NRR group Forests.

| Pairwise comparisons - Surface | | | |  | Pairwise comparisons - Longitude | | | |  | Pairwise comparisons - Latitude | | | |
| --- | --- | --- | --- | --- | --- | --- | --- | --- | --- | --- | --- | --- | --- |
|  |  | **W** | **p** |  |  |  | **W** | **p** |  |  |  | **W** | **p** |
| A | B | 0.611 | 0.902 |  | A | B | -0.377 | 0.962 |  | A | B | 1.97 | 0.346 |
| A | C | -0.775 | 0.848 |  | A | C | 2.592 | 0.159 |  | A | C | 2.06 | 0.311 |
| B | C | -1.664 | 0.467 |  | B | C | 3.273 | 0.054 |  | B | C | 1.40 | 0.585 |

### **Table S16a**

One-way ANOVA (non parametric) test (Kruskal-Wallis test) results for habitat surface coverage and geographic location parameters (longitude and latitude) between the 3 degrees of conservation for NRR group Steppe, heat and scrub habitats. In bold are highlighted the values that show significant differences.

| Kruskal-Wallis | | | | | | | |
| --- | --- | --- | --- | --- | --- | --- | --- |
|  | | **χ²** | | **df** | | **p** | |
| Surface |  | 0.911 |  | 2 |  | 0.634 |  |
| Longitude |  | 3.158 |  | 2 |  | 0.206 |  |
| Latitude |  | 7.411 |  | 2 |  | **0.025** |  |
|  | | | | | | | |

### **Table S16b**

Dwass-Steel-Critchlow-Fligner test results for habitat surface coverage and geographic location parameters (longitude and latitude), a pairwise comparison between the degrees of conservation in NRR group Steppe, heat and scrub habitats.

| Pairwise comparisons - Surface | | | |  | Pairwise comparisons - Longitude | | | |  | Pairwise comparisons - Latitude | | | |
| --- | --- | --- | --- | --- | --- | --- | --- | --- | --- | --- | --- | --- | --- |
|  |  | **W** | **p** |  |  |  | **W** | **p** |  |  |  | **W** | **p** |
| A | B | -0.180 | 0.991 |  | A | B | 2.010 | 0.330 |  | A | B | -3.665 | 0.026 |
| A | C | -1.382 | 0.592 |  | A | C | 2.073 | 0.308 |  | A | C | -2.363 | 0.217 |
| B | C | -1.173 | 0.685 |  | B | C | 0.981 | 0.767 |  | B | C | -0.435 | 0.949 |

### **Table S17a**

One-way ANOVA (non parametric) test (Kruskal-Wallis test) results for habitat surface coverage and geographic location parameters (longitude and latitude) between the 3 degrees of conservation for NRR group Rocky and dune habitats. In bold are highlighted the values that show significant differences.

| Kruskal-Wallis | | | | | | | |
| --- | --- | --- | --- | --- | --- | --- | --- |
|  | | **χ²** | | **df** | | **p** | |
| Surface |  | 11.82 |  | 2 |  | **0.003** |  |
| Longitude |  | 1.78 |  | 2 |  | 0.412 |  |
| Latitude |  | 6.39 |  | 2 |  | **0.041** |  |
|  | | | | | | | |

### **Table S17b**

Dwass-Steel-Critchlow-Fligner test results for habitat surface coverage and geographic location parameters (longitude and latitude), a pairwise comparison between the degrees of conservation in NRR group Rocky and dune habitats. In bold are highlighted the values that show significant differences.

| Pairwise comparisons - Surface | | | |  | Pairwise comparisons - Longitude | | | |  | Pairwise comparisons - Latitude | | | |
| --- | --- | --- | --- | --- | --- | --- | --- | --- | --- | --- | --- | --- | --- |
|  |  | **W** | **p** |  |  |  | **W** | **p** |  |  |  | **W** | **p** |
| A | B | -2.08 | 0.306 |  | A | B | 1.4372 | 0.567 |  | A | B | -0.0912 | 0.998 |
| A | C | -4.52 | **0.004** |  | A | C | 0.0876 | 0.998 |  | A | C | -2.6637 | 0.143 |
| B | C | -3.44 | **0.040** |  | B | C | -1.6601 | 0.469 |  | B | C | -3.3130 | **0.050** |
